# Draper/CED-1 Mediates an Ancient Damage Response to Control Inflammatory Blood Cell Migration In Vivo

**DOI:** 10.1016/j.cub.2015.04.037

**Published:** 2015-06-15

**Authors:** Iwan Robert Evans, Frederico S.L.M. Rodrigues, Emma Louise Armitage, Will Wood

**Affiliations:** 1 Department of Infection and Immunity and Bateson Centre, University of Sheffield, Firth Court, Western Bank, Sheffield S10 2TN, UK; 2 School of Cellular and Molecular Medicine, Faculty of Medical Sciences, University of Bristol, Medical Sciences Building, University Walk, Bristol BS8 1TD, UK

## Abstract

Tissue damage leads to a robust and rapid inflammatory response whereby leukocytes are actively drawn toward the wound. Hydrogen peroxide (H_2_O_2_) has been shown to be an immediate damage signal essential for the recruitment of these inflammatory blood cells to wound sites in both *Drosophila* and vertebrates [1, 2]. Recent studies in zebrafish have shown that wound-induced H_2_O_2_ is detected by the redox-sensitive Src family kinase (SFK) Lyn within the responding blood cells [3]. Here, we show the same signaling occurs in *Drosophila* inflammatory cells in response to wound-induced H_2_O_2_ with mutants for the Lyn homolog Src42A displaying impaired inflammatory migration to wounds. We go on to show that activation of Src42A is necessary to trigger a signaling cascade within the inflammatory cells involving the ITAM domain-containing protein Draper-I (a member of the CED-1 family of apoptotic cell clearance receptors) and a downstream kinase, Shark, that is required for migration to wounds. The Src42A-Draper-Shark-mediated signaling axis is homologous to the well-established SFK-ITAM-Syk-signaling pathway used in vertebrate adaptive immune responses. Consequently, our results suggest that adaptive immunoreceptor-signaling pathways important in distinguishing self from non-self appear to have evolved from a more-ancient damage response. Furthermore, this changes the role of H_2_O_2_ from an inflammatory chemoattractant to an activator signal that primes immune cells to respond to damage cues via the activation of damage receptors such as Draper.

## Highlights

• Draper works downstream of H_2_O_2_ to drive macrophage migration to wounds in *Drosophila*
• Draper’s ITAM domain is critically required for migration of macrophages to wounds
• Draper’s ITAM and NPXY motifs have separable functions in *Drosophila* macrophages
• SFK-ITAM-Syk signaling regulates an ancient damage response in *Drosophila*

## Teaser

Evans et al. reveal that Draper functions in concert with a Src family kinase (Src42A) and a Syk homolog (Shark) to regulate migration of macrophages to sites of damage in *Drosophila*. This function critically depends upon Draper’s immunoreceptor tyrosine-based activation motif and is separable from Draper’s canonical role in clearance of dying cells.

## Results and Discussion

**Figure 1:**
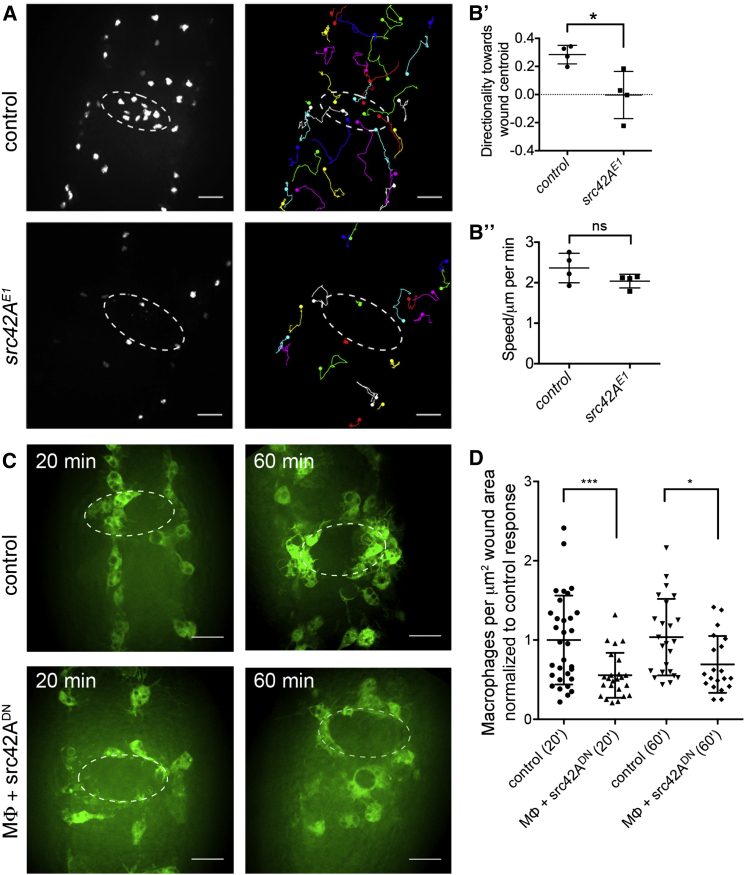
*src42A* Is Required Specifically and Autonomously for Macrophage Wound Responses in *Drosophila* Embryos (A) Stills and trajectories of red-stinger-labeled macrophages taken at 20 min after wounding from movies of inflammatory responses to wounds in control and *src42A*^*E1*^ mutant embryos. (B) Scatterplots of directionality toward the center of wounds and average speed (per macrophage, per embryo) during wound responses show that macrophages in *src42A*^*E1*^ mutants essentially ignore wounds (B′) but that their ability to move at normal speeds is unaffected (B′′). (C) Representative stills of GFP-labeled macrophages (green) at wound sites at 20 and 60 min after wounding in control embryos and embryos expressing a dominant-negative version of Src42A in macrophages. (D) Scatterplot of wound responses shows numbers of macrophages per μm^2^ of wound area normalized according to control averages. Scale bars represent 20 μm. Central lines and error bars on scatterplots represent mean and SD, respectively; ns, not significant; ^∗^p < 0.05 and ^∗∗∗^p < 0.001 via Mann-Whitney test (B) or one-way ANOVA followed by Sidak’s multiple comparisons test (D); Mϕ, macrophages; white ovals depict wound edges. See also Figures S1 and S3 and Movie S1.

Because H_2_O_2_ is an evolutionarily conserved, wound-induced damage signal and has been shown to be detected by the redox-sensitive Src family kinase (SFK) Lyn in zebrafish neutrophils [3], we began by investigating whether Lyn’s closest relative played a similar role in the recruitment of inflammatory cells to wounds in *Drosophila* embryos. The SFK most closely related to zebrafish Lyn in *Drosophila* is Src42A, and the critical redox-sensitive cysteine residue necessary for H_2_O_2_ detection in the fish (C466) is conserved in Src42A but is absent from the remaining SFKs and related non-receptor tyrosine kinases (*src62B*, *abl*, and *btk29A*) [3]. Live imaging of macrophage (hemocyte) responses to laser-induced epithelial wounds in *src42A*^*E1*^ mutant embryos revealed that these inflammatory cells essentially ignored such wounds, exhibiting directionalities close to zero (Figures 1A–1B′; Movie S1). Immunostaining revealed a strong loss of Src activity in these mutant embryos (Figure S1A), and the *src42A*^*E1*^ wound recruitment defect was phenocopied when placed in a heteroallelic combination with a *src42A*^*myri*^ loss-of-function allele (Figures S1B′ and S1B′′), revealing this inflammatory deficit as specific to *src42A*. Developmental dispersal of macrophages (Figures S1C and S1D) and migration speeds of *src42A* mutant macrophages following injury (Figure 1B′′) were indistinguishable from controls, suggesting that their migratory machinery remains intact; therefore defective motility is unlikely to underlie the failure of these cells to respond to wounds. Furthermore, *src42A* does not appear necessary for specification or proliferation of *Drosophila* macrophages, demonstrating a specific role in these cells for *src42A* in wound recruitment (Figures S1C and S1D). In order to test whether the wound recruitment defect observed in *src42A* mutants is due to a macrophage-specific requirement for Src42A, we expressed a dominant-negative version of Src42A (Src42A^DN^) [4] specifically in macrophages and assessed their ability to respond to wounds. Disrupting Src42A function in this way was sufficient to impair inflammatory recruitment following wounding, demonstrating a cell-autonomous function for Src42A (Figures 1C and 1D).

Expression of Src42A^DN^ within macrophages did not alter overall cellular morphology, though Src42A^DN^ macrophages exhibited slightly larger spread areas in vivo (Figures S1E′ and S1E′′) and migrated marginally faster than controls (data not shown). In contrast, macrophages in *src42A* zygotic mutants appeared slightly smaller when visualized live (Figure S1F), potentially reflecting non-macrophage autonomous effects and an altered in vivo environment, which presumably explains the stronger inflammatory defects in these embryos compared to those observed in Src42A^DN^ experiments; for example *src42A* has a role in epidermal responses to injury [5].

In summary, *src42A* appears to have a specific role in governing macrophage responses to injury in *Drosophila* embryos and this function is consistent with the related role of zebrafish Lyn in neutrophils [3], where it operates as a redox sensor to alert blood cells to the presence of wound-induced H_2_O_2_.

**Figure 2:**
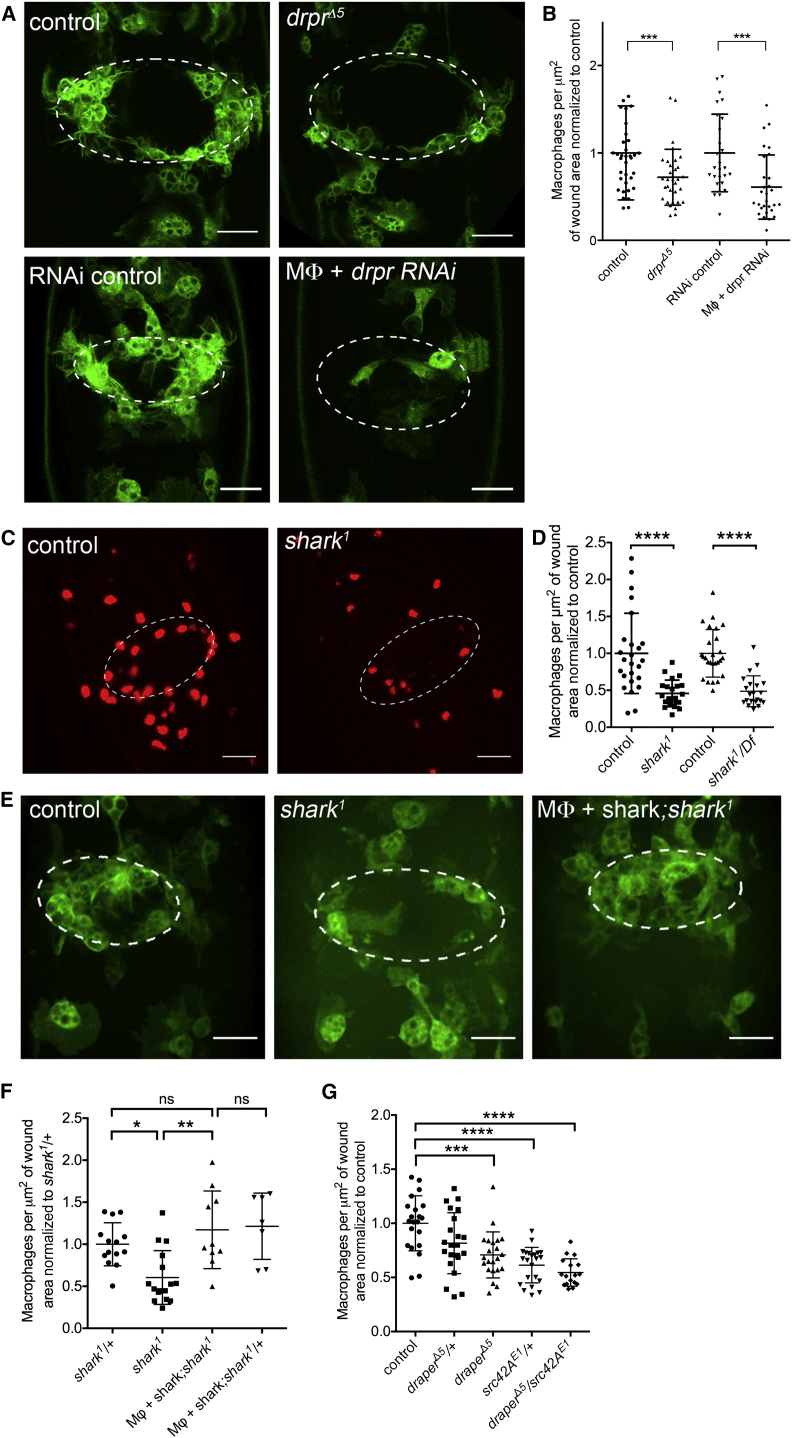
Cell-Autonomous Requirement for *draper* and *shark* and Genetic Interaction between *src42A* and *draper* during Macrophage Recruitment to Wounds (A) Representative stills of GFP-labeled macrophages (green) at wound sites at 60 min after wounding in control and *draper*^*Δ5*^ mutant embryos or embryos in which macrophages express a *draper* RNAi construct. (B) Scatterplot of wound responses shows numbers of macrophages per μm^2^ of wound area at 60 min normalized according to control averages. (C) Representative stills of nuclear-red-stinger-labeled macrophages at 60 min after wounding reveal that embryos with a null mutation in *shark* have a strong reduction in macrophage recruitment to wounds compared to controls. (D) Scatterplot of wound responses at 60 min reveals defective macrophage recruitment in *shark*^*1*^ and *shark*^*1*^*/Df* embryos. (E) Representative stills of GFP-labeled macrophages (green) at 60 min post-wounding reveal that overexpression of *shark* in macrophages rescues *shark*^*1*^ mutant wound response defects. (F) Scatterplot of wound responses shows rescue of macrophage responses at 60 min with macrophage-specific expression of shark in *shark*^*1*^ mutant background, compared to *shark*^*1*^ mutants. (G) Scatterplot of wound responses in *src42A*^*E1*^*/draper*^*Δ5*^ transheterozygotes reveals a genetic interaction of these genes during recruitment of macrophages to wounds. White ovals display wound margin; central lines and error bars on scatterplots represent mean and SD, respectively; ^∗^p < 0.05, ^∗∗^p < 0.01, ^∗∗∗^p < 0.001, and ^∗∗∗∗^p < 0.0001 according to Mann-Whitney test (B, D, and G) or one-way ANOVA with Sidak’s multiple comparisons test (F); scale bars represent 20 μm (A). See also Figure S2 and S3.

What signaling is occurring downstream of Src42A following the detection of H_2_O_2_? In *Drosophila* glia responding to degenerating axons, *src42A* interacts genetically with the *Drosophila* CED-1 homolog *draper*, where a tyrosine in Draper’s ITAM (immunoreceptor tyrosine-based activation motif) domain is critical for these responses [6]. Src42A can phosphorylate Draper at this residue in vitro, and this results in the recruitment of a Syk-related kinase called Shark [6]. In glia, *shark* also genetically interacts with *src42A* and *draper* [6], revealing a tri-partite signaling axis evocative of the well-established SFK-ITAM-Syk-signaling paradigm employed in adaptive immune responses in vertebrates [7]. We therefore wondered whether detection of wound-induced H_2_O_2_ by Src42A in macrophages might trigger the same signaling pathway and be important for their migration to wounds. Live imaging macrophage responses to wounds in *draper* mutants revealed that these inflammatory migrations are severely impaired with fewer macrophages present at the wound 1 hr after wounding (Figures 2A and 2B). To test whether *draper* was required cell autonomously in macrophages, we used RNAi-mediated knockdown: this approach efficiently depleted *draper-I* levels in macrophages as demonstrated by qPCR for *draper-I* transcripts in FACS-sorted macrophages (Figure S2A), and overexpression of this construct also led to loss of Draper protein in stage 15/16 embryos (Figures S2B′ and S2B′′). RNAi-mediated knockdown of *draper* specifically in macrophages led to the same reduction in inflammatory cells at wounds, demonstrating a macrophage-specific requirement for Draper in mediating efficient inflammatory responses to damage (Figures 2A and 2B). We next wanted to determine whether the third participant of the well-established SFK-ITAM-Syk immune signaling pathway was involved in this innate response to damage in vivo: live imaging of macrophage responses to laser wounds in embryos mutant for the Syk homolog *shark* showed that these also have an impaired ability to raise an inflammatory response, with less macrophages arriving at wounds 1 hr post-wounding (Figures 2C, 2D, and S2C). Importantly, we were able to demonstrate specificity and a cell-autonomous role for Shark by re-expression of Shark in macrophages within a *shark*^*1*^ mutant background, an approach that rescues wound responses to control levels (Figures 2E and 2F). A reduction in macrophage numbers does not appear to explain the wound-recruitment phenotypes, because both local and total numbers of macrophages appear unaffected in *draper*, *src42A*, and *shark* mutants (Figures S1C, S1D, and S3). To confirm *src42A* and *draper* operate in the same genetic pathway, we wounded transheterozygous *src42A/draper* mutant embryos and compared them to controls. The resulting wounds showed a reduction in inflammatory cells present at the wound sites when *src42A*^*E1*^*/+* heterozygotes were compared with *src42A*^*E1*^*/draper*^*Δ5*^ transheterozygotes, suggestive of a genetic interaction (Figure 2G). Taken together, these results suggest that a Src42A-Draper-Shark-signaling axis is critical for the efficient recruitment of inflammatory macrophages to wounds in vivo.

**Figure 3:**
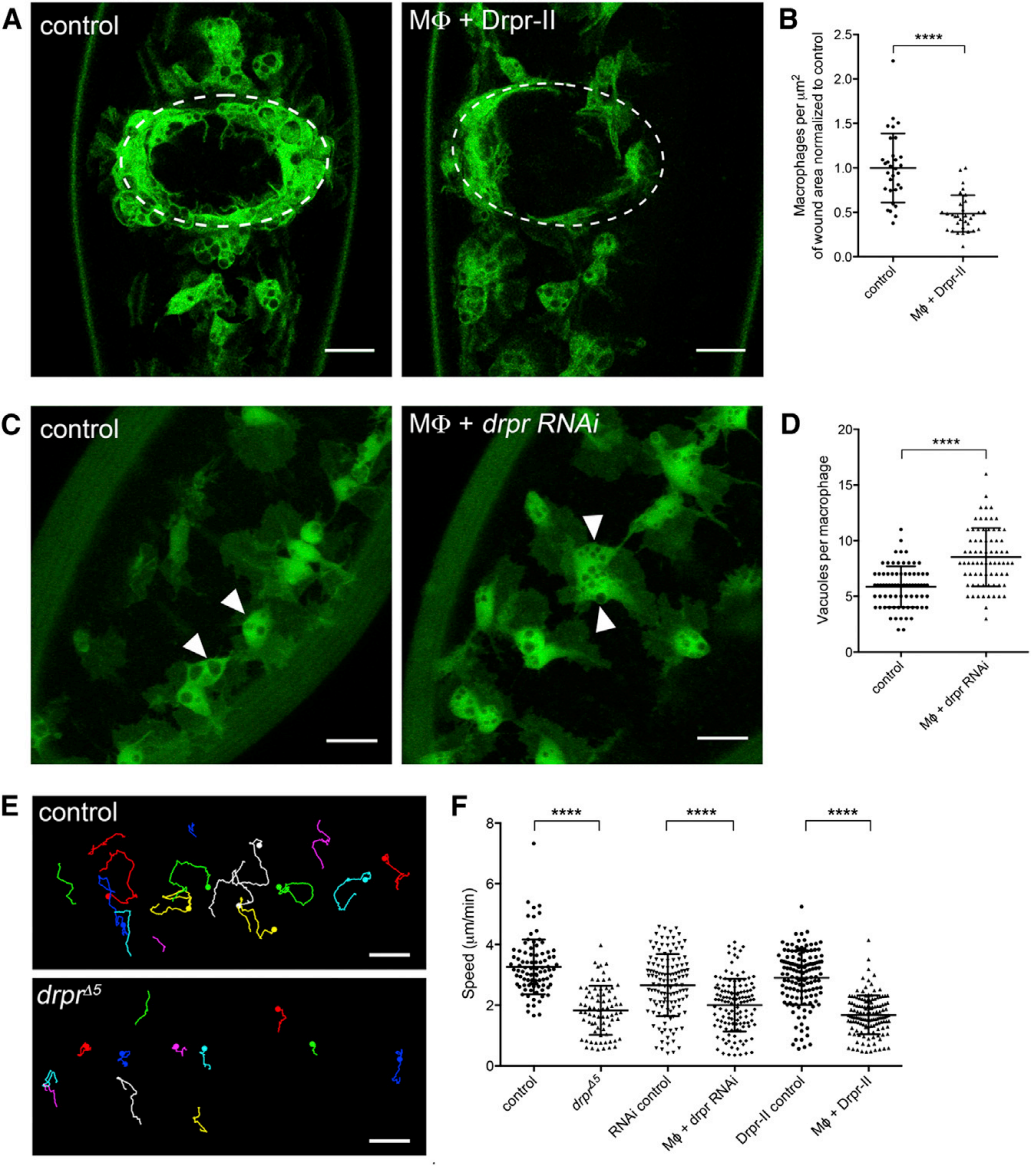
Draper Signaling Is Necessary in Macrophages for Normal Wound Responses, Apoptotic Cell Processing, and Basal Motility (A) Representative stills of GFP-labeled macrophages (green) at wound sites at 60 min after wounding showing a reduction in immune cell recruitment in embryos in which macrophages express Draper-II, compared to controls; white ovals denote wound outlines. (B) Scatterplot of wound responses shows defective recruitment of macrophages upon Draper-II expression at 60 min post-wounding. (C) Stills of GFP-labeled macrophages following macrophage-specific RNAi-mediated knockdown of Draper results in increased vacuolation of these cells, consistent with apoptotic corpse processing defects. (D) Scatterplot showing increase in number of vacuoles per macrophage on RNAi-mediated knockdown of Draper in macrophages; >30 macrophages from greater than ten embryos analyzed. (E) Representative macrophage tracks taken from 30-min movies of macrophages migrating in control and *draper* mutant embryos at stage 15, showing a reduction in basal motility in the latter; dots indicate final position of each macrophage—macrophages that leave the plane of focus during the movie terminate without a dot. (F) Scatterplot of basal motility speeds per macrophage from tracks taken from greater than four movies per genotype. Loss of *draper* function or macrophage-specific expression of an RNAi construct targeting Draper or overexpression of Draper-II reduces the speed of macrophage basal motility at stage 15. Central lines and error bars on scatterplots represent mean and SD, respectively; ^∗∗∗∗^p < 0.0001 via the Mann-Whitney test; scale bars represent 20 μm (A and C) or 25 μm (E). See also Figure S2 and S3.

During the late stages of glial responses to axonal injuries, an alternative splice variant, Draper-II, becomes highly upregulated. Rather than an ITAM, the cytoplasmic domain of Draper-II contains an ITIM (immunoreceptor tyrosine inhibitory motif) [8]. Draper-II uses its ITIM to attenuate Draper-I signaling via the recruitment of a phosphatase, Corkscrew, which dephosphorylates Shark [8]. We found that macrophage-specific expression of Draper-II also impaired inflammatory migration to laser-induced epithelial wounds in *Drosophila* embryos (Figures 3A and 3B), further demonstrating a role for Draper signaling in wound responses and highlighting the importance of the ITAM-containing intracellular domain of the Draper-I isoform in this process. ITAMs are found in many mammalian immune receptors involved in adaptive immune responses, such as B cell and T cell receptors, which, as per Draper, can be directly phosphorylated by SFKs [6, 7].

**Figure 4:**
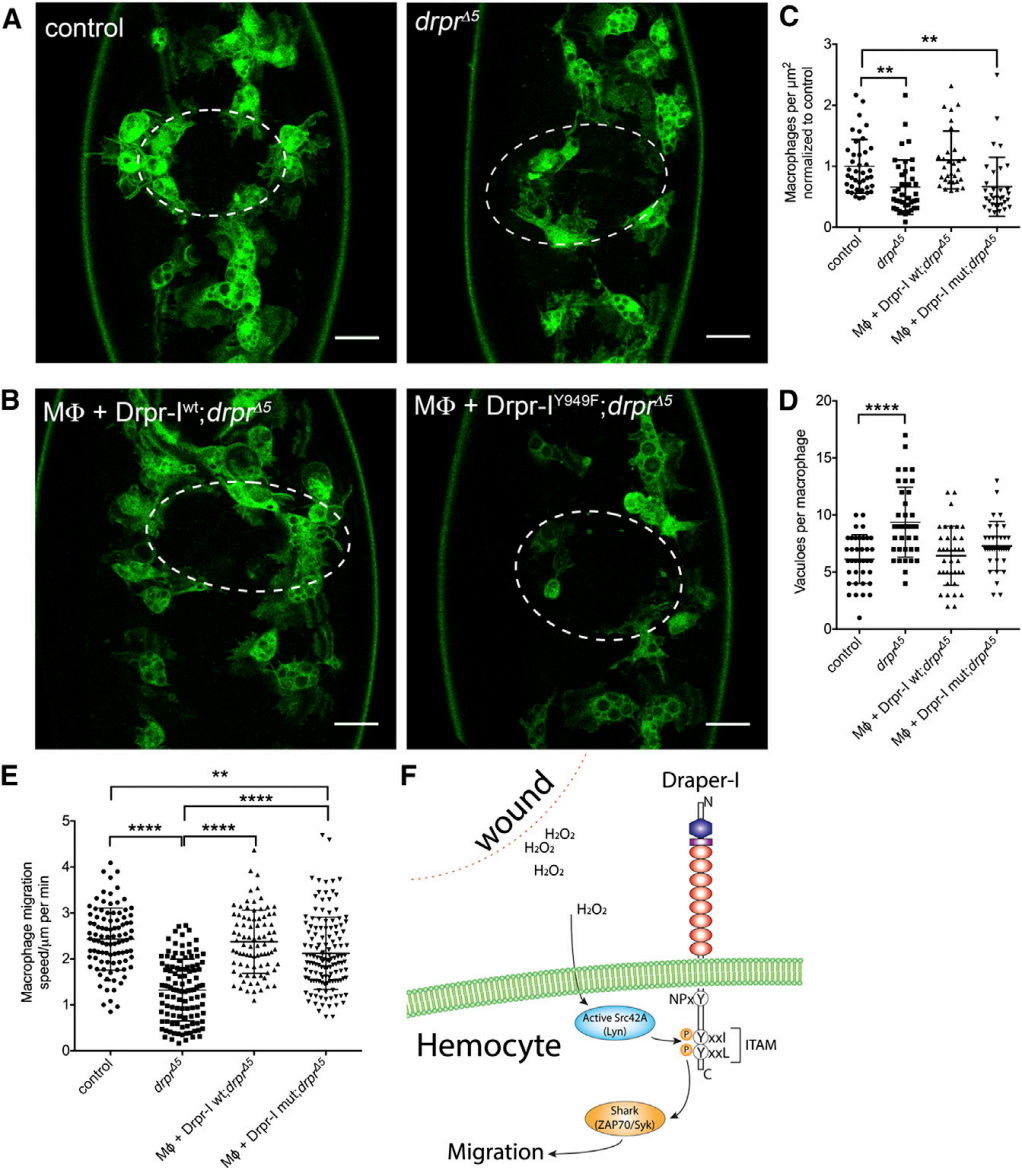
Draper’s ITAM Domain Is Specifically Required for Macrophage Chemotactic Migration toward Wounds (A and B) Representative stills of GFP-labeled macrophages (green) at wound sites at 60 min after wounding in control and *draper* mutant embryos (A) or *draper* mutants with macrophage-specific re-expression of either wild-type Draper-I (MΦ + Drpr-I^WT^;*drpr*^*Δ5*^) or a mutant form of Draper-I lacking a crucial tyrosine residue in its ITAM domain (MΦ + Drpr-I^Y949F^;*drpr*^*Δ5*^; B); white ovals denote wound outlines. (C) Scatterplots showing rescue of *draper* mutant wound responses on macrophage-specific re-expression of Draper-I^WT^ and a failure of Draper-I^Y949F^ to rescue these responses at 60 min post-wounding. (D and E) In contrast, the number of vacuoles per macrophage (D; >30 macrophages at the midline analyzed in greater than ten embryos) and the wandering macrophage migration speeds at stage 15 (E; greater than or equal to five movies per genotype) were rescued by expression of either Draper-I^WT^ or Draper-I^Y949F^. (F) Schematic illustrating the role of the Src-Draper-Shark-signaling axis in directing inflammatory responses in the *Drosophila* embryo. Central lines and error bars on scatterplots represent mean and SD, respectively; ^∗∗^p < 0.01 and ^∗∗∗∗^p < 0.0001 according to one-way ANOVA with Sidak’s multiple comparisons test; scale bars represent 20 μm (A). See also Figures S3 and S4.

Taken together, these results demonstrate a requirement for SFK-ITAM-Syk signaling in innate immune cell inflammatory responses to damage-induced H_2_O_2_ and places the ITAM-containing Draper-I variant at the center of this damage-induced signaling cascade. However, *draper* encodes a homolog of the *C. elegans* apoptotic cell clearance receptor CED-1 [9] and has been shown to play a role in the detection and/or the processing of apoptotic debris in both *Drosophila* macrophages and glia [10, 11], the two predominant phagocytes within developing *Drosophila* embryos [12–14]. Indeed, *Drosophila* embryonic macrophages actively prioritize apoptotic cells above the growth factor signals that guide their developmental migrations [2]. A role for the CED-1 family in apoptotic cell clearance appears conserved through to higher vertebrates, because Jedi-1 and MEGF10, the clearest homologs of Draper in mammals, are also involved in the removal of apoptotic cells [15, 16]. Consistent with a role in the efficient processing of engulfed apoptotic debris [11], we found that *draper* mutant and draper RNAi-expressing macrophages appeared vacuolated, containing increased numbers of apoptotic corpses per cell compared to controls (Figures 3C, 3D, and 4D). We have previously shown that efficient processing of engulfed apoptotic corpses is critical for normal macrophage migration to occur [17], and similarly, we were able to observe a reduction in basal migration speeds of macrophages in *draper* mutant embryos or upon macrophage-specific expression of either Draper RNAi or Draper-II (Figures 3E and 3F).

Draper’s role in the engulfment and degradation of apoptotic and axonal debris requires the adaptor Ced-6 [18–20], the recruitment of which depends on the presence of an NPXY motif within the cytoplasmic domain of Draper [20, 21]. Our findings, however, suggest that Draper’s role in wound detection may be more reliant on its ITAM domain, because despite the presence of an NPXY motif in the Draper-II isoform, expression of Draper-II antagonized wound recruitment. To attempt to separate Draper’s role in clearance and migration, we asked whether expression of a form of Draper-I that lacked the Src phosphorylation site on its ITAM domain (Drpr-I^Y949F^) [6] was able to rescue the ability of macrophages either to migrate to a wound or process engulfed apoptotic corpses in a *draper*-null mutant background. Crucially, both Draper-I constructs can be expressed at comparable levels, including when expressed in macrophages in a *draper* mutant background (Figures S4A and S4B), while localization and expression levels were very similar in overexpressing macrophages cultured in vitro (Figures S4C′ and S4C′′). This suggests there are no intrinsic differences in expression levels as a result of transgene insertion sites, nor are there differences in protein localization between constructs that might undermine this approach. The Y949F mutation strongly perturbs Draper-Shark interactions in vitro [6], and we found that, whereas expression of full-length Draper-I^WT^ in *draper* mutant macrophages was sufficient to rescue their ability to launch an inflammatory response to wounds, expression of the version lacking the Src phosphorylation site was not (Figures 4A–4C). However, the same mutated version of Draper rescued vacuolation defects (Figure 4D) and basal migration rates (Figure 4E) as robustly as the expression of full-length Draper-I^WT^, demonstrating that, whereas its ITAM domain is critical for Draper’s function in macrophage recruitment to wounds, the ITAM domain is dispensable for its role in apoptotic corpse processing, which instead may rely more heavily on the NPXY motif.

Our findings demonstrate a novel role for Draper in the innate immune inflammatory response to wounds and place it downstream of the early damage cue H_2_O_2_. Our results suggest that H_2_O_2_ production at wounds is detected by Src42A acting as a redox sensor within macrophages and that this then triggers the phosphorylation of Draper-I on its ITAM domain and the downstream recruitment and activation of the kinase Shark (Figure 4F). SFK-ITAM-Syk signaling is a well-established immune-signaling pathway used in the mammalian adaptive immune response during B cell and T cell signaling.

Our results suggest that this adaptive immune-signaling pathway important in distinguishing self from non-self appears to have evolved from a more-ancient damage response and changes the role of H_2_O_2_ from an inflammatory chemoattractant to an activator signal that potentially primes immune cells to respond to damage cues via the activation of damage receptors such as Draper and possibly other ITAM domain-containing proteins.

## Experimental Procedures

### *Drosophila* Stocks and Genetics

For a full list of genotypes used in this study, see Table S1. Crosses were performed in laying cages with apple juice agar plates and flies left to lay overnight at 22°C with embryos collected the following day, with the exception of the Src42A^DN^ and *draper* RNAi overexpression experiments, in which embryos were laid at 29°C overnight. *Drosophila* mutants and transgenic lines were obtained from the Bloomington Stock Centre unless otherwise stated (see Table S2 for details). Further information on the lines used can also be found in Flybase [22]. Inserts of *srp-GAL4* [23], *crq-GAL4*, and *pxn-GAL4* [24] were recombined with either *UAS-EGFP* or *UAS-nuclear red stinger* in order to track macrophage movement and morphology, whereas *da-GAL4* [25] was used to drive ubiquitous expression from UAS transgenes. The following mutants and transgenes were used in this study: *w*^*1118*^ (as a “wild-type” control background), *src42A*^*E1*^ [26], *src42A*^*K10108*^ [27, 28], *src42A*^*myri*^ [29], *shark*^*1*^ [30], the *shark*-deleting deficiency *Df*(*2L*)*BSC434* [31], *draper*^*Δ5*^ [9], *UAS-draper RNAi*^HM501623^ [32], *UAS-src42A*^*DN*^ [4], *UAS-draper-I*^*WT*^ [33], *UAS-shark-2* [30], *UAS-draper-I*^*Y949F*^ line 24127-5 [8], and *UAS-draper-II* line 03529-6 [8]. Mutants were discriminated through selection against *CTG*, *TTG*, or *GAL4*-independent fluorescent balancer chromosomes [34, 35].

### Live Imaging of *Drosophila* Macrophages

For all live imaging experiments, stage 15 embryos were collected from overnight apple juice agar plates and mounted on slides in a minimal volume of 10S Voltalef oil (VWR), following dechorionation in bleach for 1 min and extensive washing in water. All imaging was carried out at room temperature. For dynamic imaging of wound responses, epithelial wounds were induced using a nitrogen-pumped Micropoint ablation laser tuned to 435 nm (Andor Technologies), as per Razzell et al. [36]. EGFP or nuclear-red-stinger-expressing macrophages were followed at 30-s intervals for 20 min post-wounding using a 40× oil immersion objective lens on a PerkinElmer UltraView spinning disc system. Basal migration speeds were analyzed by making time-lapse movies of macrophages specifically expressing either nuclear red stinger or EFGP in embryos on the ventral side of the embryo at stage 15 of development using a Leica LSM510 confocal and a 40× oil immersion objective. Movies were as per Evans et al. [17], with z stacks collected every 30 s for 20 min. Wound responses were also quantified at 20 and 60 min post-wounding (numbers of macrophages per μm^2^ of wound, normalized to the average wound response of the control). For a detailed description of image processing and analysis, see the Supplemental Experimental Procedures.

## Author Contributions

I.R.E., F.S.L.M.R., and W.W. conceived and designed the experiments; I.R.E., F.S.L.M.R., and E.L.A. performed the experiments; and I.R.E., F.S.L.M.R., E.L.A., and W.W. wrote the paper.

## Supplemental Information

Document S1. Supplemental Experimental Procedures, Figures S1–S4, and Tables S1 and S2

Movie S1. Macrophages in *src42A*^*E1*^ Mutants Fail to Respond to Epithelial Wounds, Related to Figure 1 Twenty-minute movies showing nuclear-red-stinger-labeled macrophages and their tracks in stage 15 embryos immediately after laser wounding in control (left panel) and *src42A*^*E1*^ mutant embryos (right panel). Macrophages fail to respond to the injury appropriately in the *src42A*^*E1*^ mutant, making no net progress towards the wound. Wounds are indicated by white ovals.

Document S2. Article plus Supplemental Information
