# Supplementary material for: Draper/CED-1 Mediates an Ancient Damage Response to Control Inflammatory Blood Cell Migration In Vivo
Source: Curr Biol. 2015 Jun 15;25(12):1606–12. doi: 10.1016/j.cub.2015.04.037 (PMC4503800; doi:10.1016/j.cub.2015.04.037)
Supplement: Document S1. Supplemental Experimental Procedures, Figures S1–S4, and Tables S1 and S2 [file mmc1.pdf]

Current Biology

Supplemental Information

Draper/CED-1 Mediates an Ancient Damage Response to Control Inflammatory Blood Cell Migration In Vivo

Iwan Robert Evans, Frederico S.L.M. Rodrigues, Emma Louise Armitage, and Will Wood

Supplemental Data

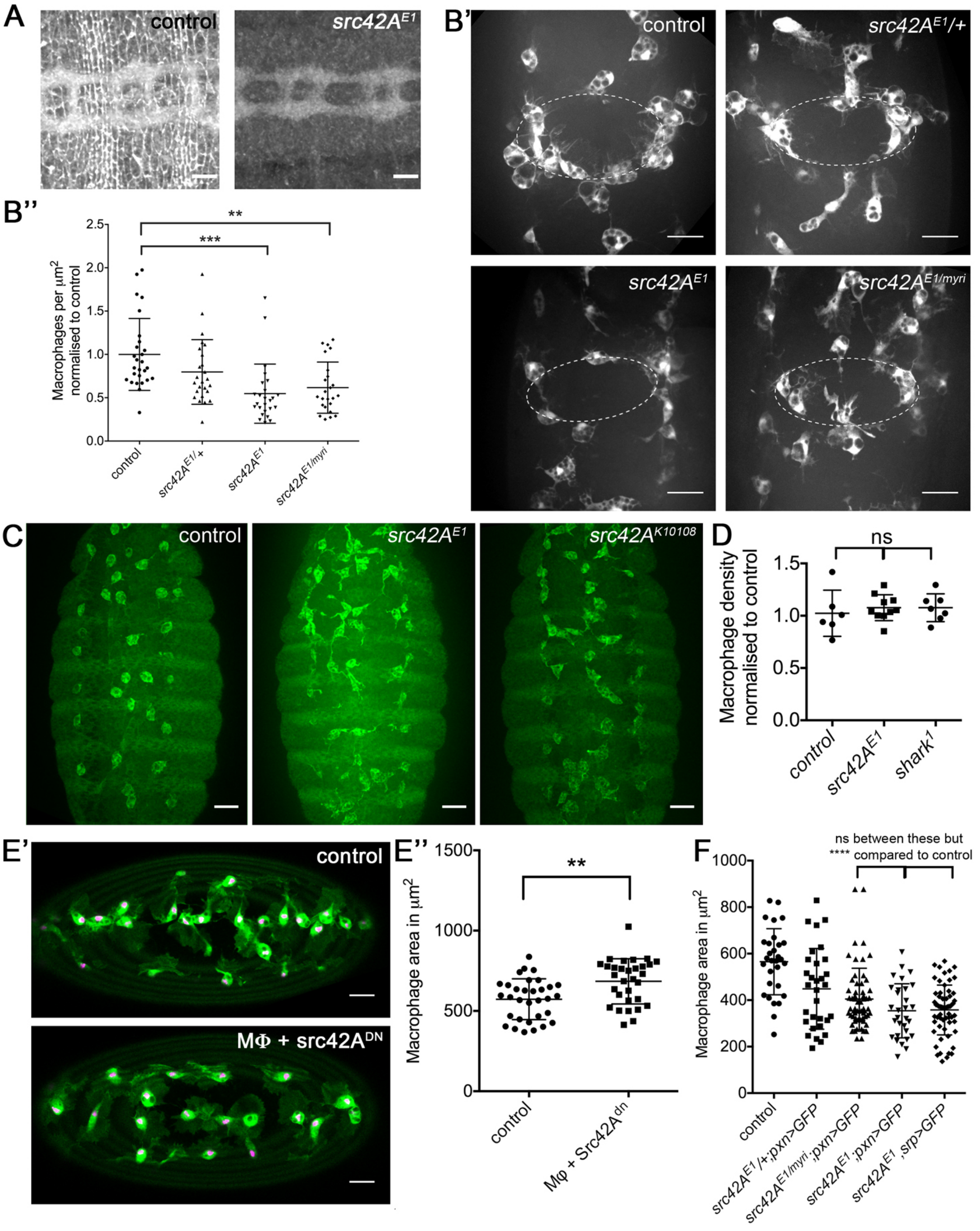

Figure S1, relates to Figure 1. *src42A* heteroallelic combinations recapitulate *src42A^{E1}* wound defects but macrophage developmental dispersal and specification appears unaffected in *src42A* mutants

To confirm loss of *src42A* function, control and *src42A^{E1}* embryos were immunostained for pSrc, showing a significant reduction in overall levels of active Src (A). Representative stills of *pxn-Gal4,UAS-GFP*-labelled macrophages at wound sites 60 min post-wounding in control,

src42A^{E1}/+ heterozygous, *src42A*^{E1} homozygous and *src42A*$^{E1/myri}$ heteroallelic embryos (B'); white ovals denote wounds. Scatterplot of wound responses reveal that heteroallelic combinations of *src42A* recapitulate *src42A*^{E1} mutant defects in wound recruitment at 60 min post-wounding (B''). Representative images of the ventral side of stage 15 embryos immunostained for Fascin (green) demonstrate that specification and developmental migration of macrophages appear normal in *src42A* mutants (C). Expression using *pxn-Gal4* as an alternative macrophage marker also appeared normal and this was used to assess local macrophage density on the ventral midline at stage 15 in control, *src42A*^{E1} and *shark*^{1} mutant embryos (D). Normal migration and marker expression suggests macrophages are correctly specified and carry out their other functions normally in the absence of *src42A*. Similarly, macrophages expressing GFP (green) and red stinger (purple) that also express Src42A^{DN} disperse and appear morphologically normal (E'), but do exhibit a small but statistically significant increase in spread area (E''). In contrast, increasingly potent combinations of *src42A* loss-of-function alleles correlated with a reduction in spread area (F). Scale bars represent 10 µm (A) or 20 µm (B'-C and E'); central lines and error bars on scatterplots represent mean and standard deviation, respectively; **, *** and **** denote $p<0.01$, $p<0.001$ and 0.0001, respectively; via one-way ANOVA with Sidak's multiple comparisons test (B'', D and F) or the Mann-Whitney test (E''); Mϕ = macrophages.

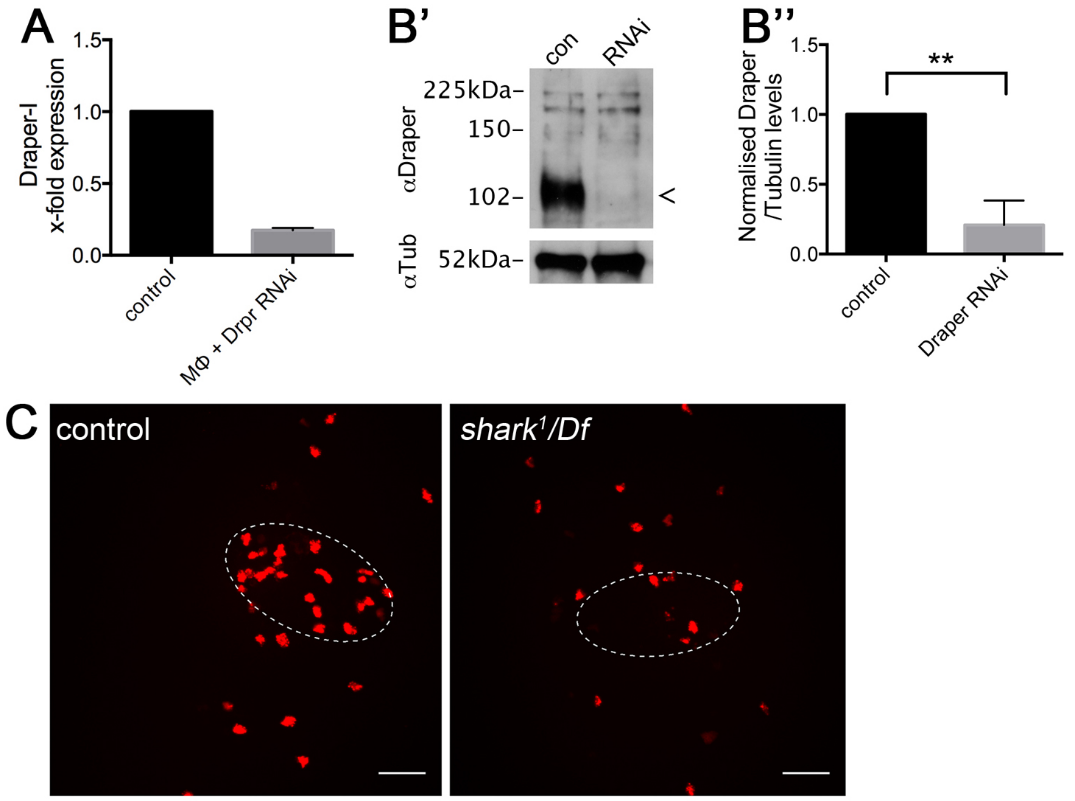

Figure S2, relates to Figures 2 and 3. Controls showing efficient knockdown of Draper via RNAi and heteroallelic *shark* loss-of-function wound images

Real-time PCR analysis of the relative expression levels of *draper-I* in embryonic stage 15 sorted macrophages was carried out following RNAi-mediated knockdown of *draper*. Draper threshold cycle (Ct) mRNA values were normalised to ribosomal protein L32 and results are presented as fold-reduction relative to control (A). Representative western blot of Draper levels in stage 15/16 embryos following *da-Gal4*-driven ubiquitous expression of the same *draper* RNAi construct; arrowhead indicates position of Draper protein (B'). Quantification of Draper knockdown, normalised to the αTubulin loading control and shown in relation to Draper-I^{wt} levels (B''; 3 biological replicates, $p<0.01$ via Student's T-test). To confirm *shark* phenotypes were not the result of a mutation elsewhere on the *shark^1*-bearing chromosome, wound recruitment was probed in embryos containing heteroallelic *shark* loss-of-function mutations. Representative images of control and *shark^1/Df* embryos showing defective recruitment of red stinger-labelled macrophages to wounds at 60 min post-wounding (C); white ovals denote wounds. N.b. quantification of this data is shown in Figure 2D. Graphs show mean and standard deviation; scale bars represent 20 μm.

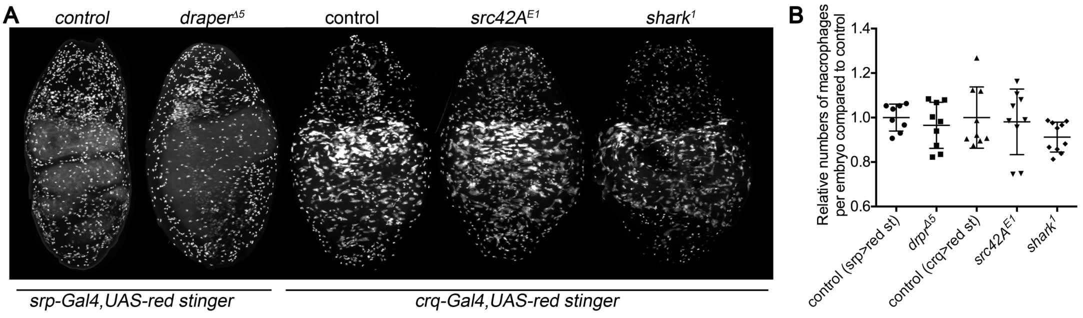

Figure S3, relates to all Figures. No differences in overall numbers of embryonic macrophages per embryo in the mutants used in this study

Representative images of squashed fly st15/16 embryos with red stinger-expressing macrophages (A). Scatterplot showing numbers of macrophages per embryo compared to controls (B); no statistically significant differences in overall numbers of macrophages was found between mutants and controls using Student's T-test (control vs *draper*$^{\Delta5}$) or a one-way ANOVA with Sidak's multiple comparisons test (control, *src42A*^{E1} and *shark*^{1}); central and outer bars represent mean and standard deviation, respectively.

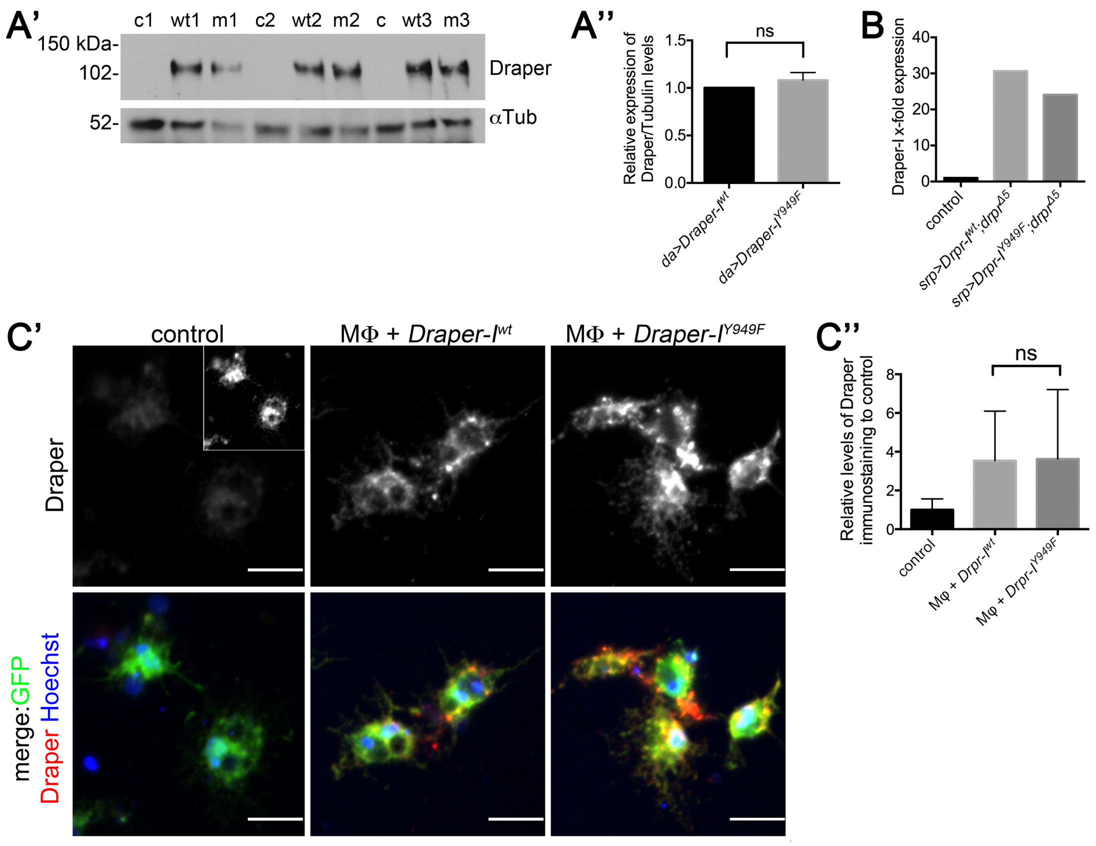

Figure S4, relates to Figure 4. Draper-I^{wt} and Draper-I^{Y949F} localise and are expressed at similar levels to each other

In order to verify comparable expression of Draper-I^{wt} (wt) and Draper-I^{Y949F} (m) protein, stage 15/16 embryos ± overexpression of each construct (via *da-Gal4*) were analysed by western blotting (A'; c = control). No difference was found between expression levels of the wild type or mutant Draper construct (A''; n=3 biological replicates with 3 blots per replicate). Real-time PCR analysis of relative expression levels of *draper-I* mRNA in whole embryos following following *srp-Gal4*-mediated *drpr-I^{wt} and drpr-I^{Y949F}* overexpression in macrophages in a *drpr^{Δ5}* mutant background. Draper threshold cycle (Ct) mRNA values were normalised to ribosomal protein L32 and results are presented as fold-reduction relative to control (B). GFP-labelled macrophages ± *srp-Gal4;crq-Gal4*-mediated expression of each construct were cultured in vitro and immunostained for Draper and GFP (C') revealing a similar subcellular localisation and similar expression levels; inset shows contrast enhanced control macrophages demonstrating a related localisation pattern for endogenous Draper. Quantification of anti-Draper immunofluorescence intensities per cell also indicated comparable expression levels for each construct (C''), though both represented a significant increase compared to

endogenous levels (>35 cells per genotype quantified from 3 biological replicates). Mϕ = macrophages; scale bars represent 10 μm; ns denotes lack of statistical significance, Student's T-test (A) or one-way ANOVA with Sidak's multiple comparisons test (C''). Bar charts show mean; error bars represent the standard deviation (A", B, C").

Supplemental Tables

Table S1, relates to experimental procedures. Genotypes of embryos used:

Figure:	Label:	Genotype:
**Fig. 1A, B**	control	*w;;crq-GAL4,UAS-nuclear red stinger*
	src42A^{E1}	*w;src42E1;crq-GAL4,UAS-nuclear red stinger*
**Fig. 1C, D**	control	*w;srp-GAL4,UAS-EGFP/+;crq-GAL4,UAS-EGFP/+*
	macrophages + src42A^{DN}	*w;srp-GAL4,UAS-gfp/+;crq-GAL4,UAS-gfp/UAS-src42A*^{DN}
**Fig. 2A, B**	control	*w;srp-GAL4,UAS-EGFP*
	draper$^{\Delta5}$	*w;srp-GAL4,UAS-EGFP;draper*$^{\Delta5}$
	RNAi control	*;srp-GAL4,UAS-GFP/+;crq-GAL4,UAS-EGFP/+*
	macrophages + *drpr RNAi*	*;srp-GAL4,UAS-GFP/UAS-draper RNAi HM501623;crq-GAL4,UAS-EGFP/+*
**Fig. 2C, D**	control	*w;;crq-GAL4,UAS-nuclear red stinger*
	shark^{1}	*w;shark*^{1}*;crq-GAL4,UAS-nuclear red stinger*
**Fig. 2D**	control	*w;;crq-GAL4,UAS-nuclear red stinger*
	shark^{1}*/Df*	*w;shark*^{1}*/Df(2L)BSC434;crq-GAL4,UAS-nuclear red stinger*
**Fig. 2E, F**	*shark*^{1}*/+*	*w;shark*^{1}*,srp-Gal4,UAS-EGFP/+*
	shark^{1}	*w;shark*^{1}*,srp-Gal4,UAS-EGFP/shark*^{1}
	macrophages + shark*;shark*^{1}	*w,UAS-shark-2/w or Y;shark*^{1}*,srp-GAL4,UAS-EGFP/shark*^{1}
	macrophages + shark*;shark*^{1}*/+*	*w,UAS-shark-2/w or Y;shark*^{1}*,srp-GAL4,UAS-EGFP/CyO ro-tLacZ*
**Fig. 2G**	control	*w;srp-GAL4,UAS-EGFP/srp-GAL4,UAS-nuclear red stinger*
	draper$^{\Delta5}$*/+*	*w;srp-GAL4,UAS-EGFP/srp-GAL4,UAS-nuclear red stinger;draper*$^{\Delta5}$*/+*
	draper$^{\Delta5}$	*w;srp-GAL4,UAS-EGFP/srp-GAL4,UAS-nuclear red stinger;draper*$^{\Delta5}$
	src42A^{E1}*/+*	*w;src42*^{E1}*/srp-Gal4,UAS-EGFP;crq-GAL4,UAS-nuclear red stinger/+*
	draper$^{\Delta5}$*/src42A*^{E1}	*w;src42*^{E1}*/srp-Gal4,UAS-EGFP;crq-GAL4,UAS-nuclear red stinger/draper*$^{\Delta5}$
**Fig. 3A, B**	control	*w;srp-GAL4,UAS-EGFP/+;crq-GAL4,UAS-EGFP/+*
	macrophages + Drpr-II	*w;srp-GAL4,UAS-GFP/UAS-draper-II;crq-GAL4,UAS-EGFP/+*
**Fig. 3C, D**	control	*w;srp-GAL4,UAS-GFP/+;crq-GAL4,UAS-EGFP/+*
	macrophages + *drpr RNAi*	*;srp-GAL4,UAS-GFP/UAS-draper RNAi HM501623;crq-GAL4,UAS-EGFP/+*
**Fig. 3E**	control	*w;srp-GAL4,UAS-nuclear red stinger*
	draper$^{\Delta5}$	*w;srp-GAL4,UAS-nuclear red stinger;draper*$^{\Delta5}$
**Fig. 3F**	control	*w;srp-Gal4,UAS-nuclear red stinger*
	draper$^{\Delta5}$	*w;srp-GAL4,UAS-nuclear red stinger;draper*$^{\Delta5}$
	RNAi control	*w;srp-GAL4,UAS-GFP/+;crq-GAL4,UAS-EGFP/+*
	macrophages + *drpr RNAi*	*;srp-GAL4,UAS-GFP/UAS-draper RNAi HM501623;crq-GAL4,UAS-EGFP/+*
	Drpr-II control	*w;srp-GAL4,UAS-EGFP/+;crq-GAL4,UAS-EGFP/+*
	macrophages + Drpr-II	*w;srp-GAL4,UAS-GFP/UAS-draper-II;crq-GAL4,UAS-EGFP/+*
**Fig. 4**	control	*w;srp-GAL4,UAS-EGFP/srp-GAL4,UAS-nuclear red stinger*
	draper$^{\Delta5}$	*w;srp-GAL4,UAS-EGFP/srp-GAL4,UAS-nuclear red stinger;draper*$^{\Delta5}$
	macrophages + Drpr-I^{wt}*;draper*$^{\Delta5}$	*w;srp-GAL4,UAS-GFP/srp-GAL4,UAS-draper-I*^{wt}*;draper*$^{\Delta5}$
	macrophages + Drpr-I^{Y949F}*;draper*$^{\Delta5}$	*w;srp-GAL4,UAS-GFP/srp-GAL4,UAS-draper-I*^{Y949F}*;draper*$^{\Delta5}$

Figure:	Label:	Genotype:
**Supplementary Fig. 1A**	control	*w*
	src42^{E1}	*w;src42A*^{E1}

Table S1 continued:

**Supplementary Fig. 1B', B''**	control	*w;;pxn-GAL4,UAS-EGFP*
	src42A^{E1}/+	*w;src42A$^{E1/+}$;pxn-GAL4,UAS-EGFP*
	src42^{E1}	*w;src42A^{E1};pxn-GAL4,UAS-EGFP*
	src42$^{E1/myri}$	*w;src42A^{E1}/src42A^{myri};pxn-Gal4,UAS-EGFP*
**Supplementary Fig. 1C**	control	*w*
	src42^{E1}	*w;src42A^{E1}*
	src42A^{K10108}	*w;src42A^{K10108}*
**Supplementary Fig. 1D**	control	*w;;pxn-GAL4,UAS-EGFP*
	src42^{E1}	*w;src42A^{E1};pxn-GAL4,UAS-EGFP*
	shark^{1}	*w;shark^{1};pxn-GAL4,UAS-EGFP*
**Supplementary Fig. 1E', E''**	control	*w;srp-GAL4,UAS-EGFP/+;crq-GAL4,UAS-nuclear red stinger/+*
	macrophages + src42A^{DN}	*w;srp-GAL4,UAS-EGFP/+;crq-GAL4,UAS-nuclear red stinger/UAS-src42A^{DN}*
**Supplementary Fig. 1F**	control	*w;;pxn-GAL4,UAS-EGFP*
	src42A^{E1}/+;pxn>GFP	*w;src42A^{E1}/+;pxn-GAL4,UAS-EGFP*
	src42A$^{E1/myri}$;pxn>GFP	*w;src42A^{E1}/src42A^{myri};pxn-Gal4,UAS-EGFP*
	src42A^{E1};pxn>GFP	*w;src42A^{E1};pxn-GAL4,UAS-EGFP*
	src42A^{E1},srp>GFP	*w;src42A^{E1},srp-GAL4,UAS-EGFP*
**Supplementary Fig. 2A**	control	*w;srp-GAL4,UAS-GFP/+;crq-GAL4,UAS-EGFP/+*
	macrophages + Draper RNAi	*;srp-GAL4,UAS-GFP/UAS-draper RNAi^{HM501623};crq-GAL4,UAS-EGFP/+*
**Supplementary Fig. 2B', B''**	con/control	*y^{1}sc*v^{1};srp-GAL4,UAS-EGFP/+;crq-GAL4,UAS-EGFP/+*
	RNAi/Draper RNAi	*y^{1}sc*v^{1};srp-GAL4,UAS-EGFP/UAS-draper RNAi^{HM501623};crq-GAL4,UAS-EGFP/+*
**Supplementary Fig. 2C**	control	*w;;crq-GAL4,UAS-nuclear red stinger*
	shark^{1}/Df	*w;shark^{1}/Df(2L)BSC434;crq-GAL4,UAS-nuclear red stinger*
**Supplementary Fig. 3A, B**	control	*w;srp-GAL4,UAS-EGFP/srp-GAL4,UAS-nuclear red stinger*
	draper$^{\Delta5}$	*w;srp-GAL4,UAS-EGFP/srp-GAL4,UAS-nuclear red stinger;draper$^{\Delta5}$*
	control	*w;;crq-GAL4,UAS-nuclear red stinger*
	src42^{E1}	*w;src42A^{E1};crq-GAL4,UAS-nuclear red stinger*
	shark^{1}	*w;shark^{1};crq-GAL4,UAS-nuclear red stinger*
**Supplementary Fig. 4A', A''**	*c1/c2/c3*	*w;;da-GAL4/+*
	wt1/wt2/wt3/*da>Draper-I^{wt}*	*w;+/UAS-Draper-I^{wt};da-GAL4/+*
	m1/mt2/m3/*da>Draper-I^{Y949F}*	*w;+/UAS-Draper-I^{Y949F};da-GAL4/+*
**Supplementary Fig. 4B**	control	*w*
	srp>Drpr-I^{wt};draper$^{\Delta5}$	*w;srp-GAL4,UAS-draper-I^{wt};draper$^{\Delta5}$*
	srp>Drpr-I^{Y949F};draper$^{\Delta5}$	*w;srp-GAL4,UAS-draper-I^{Y949F};draper$^{\Delta5}$*
**Supplementary Fig. 4C', C''**	control	*w;srp-GAL4,UAS-EGFP/+;crq-GAL4,UAS-EGFP/+*
	macrophages + *Drpr-I^{wt}*	*w;srp-GAL4,UAS-EGFP/UAS-Draper-I^{wt};crq-GAL4,UAS-EGFP/+*
	macrophages + *Drpr-I^{Y949F}*	*w;srp-GAL4,UAS-EGFP/UAS-Draper-I^{Y949F};crq-GAL4,UAS-EGFP/+*

**Supplementary Movie 1**	control	*w;;crq-GAL4,UAS-nuclear red stinger*
	src42^{E1}	*w;src42A^{E1};crq-GAL4,UAS-nuclear red stinger*

Table S2, relates to experimental procedures. Sources of alleles and transgenes:

Allele:	Chromosome:	Obtained from:
srp-GAL4	2	Katja Bruckner, UCSF, USA
crq-GAL4	3	Brian Stramer, KCL, UK
pxn-GAL4	3	Brian Stramer, KCL, UK
da-Gal4	3	Bloomington Stock Centre, USA
UAS-nuclear red stinger	2 and 3	Brian Stramer, KCL, UK
w^{1118}	X	Bloomington Stock Centre, USA
src42A^{E1}	2	Bloomington Stock Centre, USA
src42A^{K10108}	2	Bloomington Stock Centre, USA
src42A^{myri}	2	Bloomington Stock Centre, USA
shark^{1}	2	Bloomington Stock Centre, USA
Df(2L)BSC434	2	Bloomington Stock Centre, USA
draper$^{\Delta5}$	3	Paul Martin, University of Bristol, UK
UAS-draper RNAi TRiP line HM501623	2	Bloomington Stock Centre, USA
UAS-src42A^{DN}	3	David Strutt, University of Sheffield, UK
UAS-draper-I^{wt}	2	Eric Baehrecke, UMASS, USA
UAS-draper-I^{Y949F} line 24127-5	2	Marc Freeman, UMASS, USA
UAS-draper-II line 03529-6	2	Marc Freeman, UMASS, USA
UAS-shark-2	X	Marc Freeman, UMASS, USA

Supplemental Experimental Procedures

Fly genetics

Precise genotypes of the embryos used in this study can be found in Table S1, with details on the sources of alleles/transgenes contained in Table S2.

Image processing and analysis

Z-stacks of fluorescently-labelled macrophages were despeckled and assembled into maximum projections using NIH ImageJ/Fiji [S1]. Macrophage movements in wound movies or movies of basal motility were tracked using the manual tracking plugin in ImageJ. To quantify overall behaviour of macrophages in movies following laser wounding, macrophage movements were measured with respect to the center of the wound over the course of the 20 min movie. The centre (centroid) of the wound was determined from brightfield/transmitted laser light images. Tracking enabled the net distance each macrophage moved towards or away from the center of the wound over the timecourse of the movie to be determined; macrophages were followed once they were within 60 μm of the wound's center. These measurements enabled the calculation of directionality towards the center of the wound (Cartesian distance from center of the wound at t = 0 min/net distance moved with respect to

the center of the wound) for each cell analysed. Directionality per macrophage was then averaged per embryo to give an overall measure of the wound response; only movies with wounds between 1000 and 2000 µm^2 at 10 min post-wounding were used in this dynamic analysis. For movies of basal motility speed was calculated by dividing the total distance moved over course of the movie by the time taken to move that distance.

To quantify macrophage wound responses from individual timepoints (20 or 60 min post-wounding), the number of macrophages in contact with/inside the wound edge was determined from z-stacks. Wound size was determined from brightfield images in ImageJ. The number of macrophages was then divided by the wound area and the value normalised according to the appropriate control average.

Z-stacks were also used to measure vacuolation (EGFP-negative inclusions within the macrophage cell body - only macrophages that were clearly distinct from their neighbours on the ventral midline were assessed to rule out the possibility of counting vacuoles from > 1 macrophage) or macrophage density (numbers on the ventral midline/cross-sectional area of the most dorsal confocal slice through the embryo). Maximum z-projections were used to measure macrophage spread area using the lasso tool in ImageJ. As per vacuolation analyses, only individual cells lying between the edges of the ventral nerve cord that could be distinguished from their neighbours were analysed, with > 30 cells quantified from at least 5 different embryos for each genotype.

To quantify Draper expression levels in vitro, the GFP channel of maximum projections was used to draw a mask around each GFP-positive macrophage. This masked selection was then used to measure the intensity of anti-Draper staining per cell and the spread area. The integrated density (intensity or mean gray value x area) was normalised to control levels to enable the comparison of Draper expression levels. All image analysis was carried out on blinded images and identical adjustments were made to contrast and brightness across experimental groups. All statistical analysis and production of graphs was carried out using Prism 6 (GraphPad).

Immunofluorescent staining of *Drosophila* embryos and macrophages in vitro

Embryos were fixed and immunostained as per Evans et al., 2010 [S2], using rabbit anti-LacZ (1:2000, Cappel), purified mouse anti-Fascin (1:100 clone sn7C, Developmental Studies Hybridoma Bank) or rabbit anti-pSrc (1:500, Invitrogen) as primary antibodies with goat anti-rabbit-AlexaFluor488 (Molecular probes), goat anti-mouse-FITC (Jackson Immunoresearch Laboratories) or goat anti-rabbit-AlexaFluor568 (Molecular Probes) used as secondary antibodies, respectively.

To probe Draper expression 14 stage 15 embryos were disaggregated in 70 μL 10 % FBS in Schneiders medium (Sigma) and allowed to adhere to Thermanox plastic coverslips (Nunc) for 2 h at room temperature in a humidified chamber. Macrophages were then fixed for 10 min in 4 % formaldehyde (Agar Scientific) in PBS. Macrophages were then permeabilised with 0.1 % Triton-X100 in PBS for 3 min and blocked with 2 % horse serum in PBS for 45 min. GFP-expressing macrophages were then stained using mouse anti-GFP (1:100, Abcam) and rabbit anti-Draper (1:500, kind gift from Marc Freeman [S3]) for 45 min in 2 % horse serum, with primary antibodies detected via goat anti-mouse-FITC and goat anti-rabbitAlexaFluor568 (both 1:200 in 2 % horse serum/PBS for 30 min), while Hoechst/NucBlue (Molecular Probes) was added as a nuclear dye. Cells were then mounted in Mowiol under a coverslip before confocal imaging on a Nikon A1 system using a 63X oil immersion lens.

Analysis of macrophage numbers per embryo

Embryos containing red stinger-expressing macrophages were fixed in 4 % formaldehyde according to standard fixation protocols [S2] and then cleared overnight in Dabco mountant at 4°C. Embryos were then transferred to slides in a minimal volume of Dabco and flattened under a coverslip in Mowiol mountant, such that macrophages were all in the same focal plane once the Mowiol had set. Embryos were then imaged on a Leica M205FA stereomicroscope fitted with a Leica DCF365 FX camera, with panoramic images of the whole embryo stitched together using the MosaicJ plugin [S4] in ImageJ. These images were

then blinded and macrophage numbers per embryo counted using red stinger expression and nuclear morphology to identify macrophages.

Western blotting of embryo lysates

30 stage 15/16 embryos were crushed and dissolved in 60 µl 2.5X sample buffer per sample, then boiled for 10 min at 95°C, before separation via SDS-PAGE on 7.5 % pre-cast gels (Bio-Rad). Standard wet transfer techniques were used to transfer separated proteins to nitrocellulose filters, which were subsequently blocked using 5 % BSA (Sigma) in TBS-Tween before incubation overnight at 4°C in rabbit anti-Draper [S3] or rabbit anti-αTubulin (Cell Signalling Technologies), with each diluted 1:1000 in blocking solution. After washing in 1 % BSA in TBS-Tween, goat anti-rabbit-HRP secondary antibodies (Dako) were used at a dilution of 1:6000 in 1 % BSA in TBS-Tween for 90 min at room temperature. After washing in TBS-T, secondary antibodies were detected using ECL select (Amersham). Blots were scanned and quantified by normalising the integrated density of Draper bands according to the corresponding αTubulin band after background subtraction. These values were then normalised according control (Figure S2) or Draper-I^{wt} levels (Figure S4) to reveal RNAi-mediated knockdown or compare expression with that of Draper-I^{Y949F}, respectively.

Macrophage sorting and qPCR

For sorting of macrophages, 50 embryos of each genotype were disaggregated, strained using a 40 µm mesh and resuspended in 500 µL Seecoff's buffer. Suspensions were then sorted using an Influx™ cell sorter with a 488nm Laser (BD Biosciences). Non-fluorescent embryos were used to set sort gates.

For quantification of RNAi-mediated knockdown of *draper-I* mRNA was extracted from sorted macrophages using RNAqueous-Micro Total RNA Isolation Kit (Ambion). For quantification of *drpr-I* expression in *drpr*$^{\Delta 5}$ mutant rescue experiments 50 embryos of each genotype were crushed in QIAzol Lysis Reagent (Qiagen) and RNA purified using an RNeasy Mini Kit (Qiagen). In both experiments RNA samples were then treated with DNase and RNA quantified using a NanoDrop 2000c (Thermo Scientific). Equal quantities of RNA were then

reverse transcribed using SuperScript III First-Strand Synthesis System (Invitrogen). Relative quantification of gene expression was carried out on a LightCycler 480 Real-Time PCR machine (Roche). The following Taqman assays (Applied Biosystems) were used: (i) Ribosomal protein L32 (ABI pre-made assay Dm02151827_g1) (ii) Draper-I custom assay, F-primer, TGTGATCATGGTTACGGAGGAC; R-primer, CAGCCGGGTGGGCAA; probe, CGCCTGCGATATAA as described in Logan et al., (2012) [S5]. Analysis was performed on $2^{-(\Delta\Delta Ct)}$ values.

Supplemental References

S1. Schindelin, J., Arganda-Carreras, I., Frise, E., Kaynig, V., Longair, M., Pietzsch, T., Preibisch, S., Rueden, C., Saalfeld, S., Schmid, B., et al. (2012). Fiji: an open-source platform for biological-image analysis. Nature methods 9, 676-682.

S2. Evans, I.R., Hu, N., Skaer, H., and Wood, W. (2010). Interdependence of macrophage migration and ventral nerve cord development in Drosophila embryos. Development 137, 1625-1633.

S3. Freeman, M.R., Delrow, J., Kim, J., Johnson, E., and Doe, C.Q. (2003). Unwrapping glial biology: Gcm target genes regulating glial development, diversification, and function. Neuron 38, 567-580.

S4. Thevenaz, P., and Unser, M. (2007). User-friendly semiautomated assembly of accurate image mosaics in microscopy. Microscopy research and technique 70, 135-146.

S5. Logan, M.A., Hackett, R., Doherty, J., Sheehan, A., Speese, S.D., and Freeman, M.R. (2012). Negative regulation of glial engulfment activity by Draper terminates glial responses to axon injury. Nature neuroscience 15, 722-730.
