# Supplementary figures and images for: Draper/CED-1 Mediates an Ancient Damage Response to Control Inflammatory Blood Cell Migration In Vivo
Source: Curr Biol. 2015 Jun 15;25(12):1606–12. doi: 10.1016/j.cub.2015.04.037 (PMC4503800; doi:10.1016/j.cub.2015.04.037)

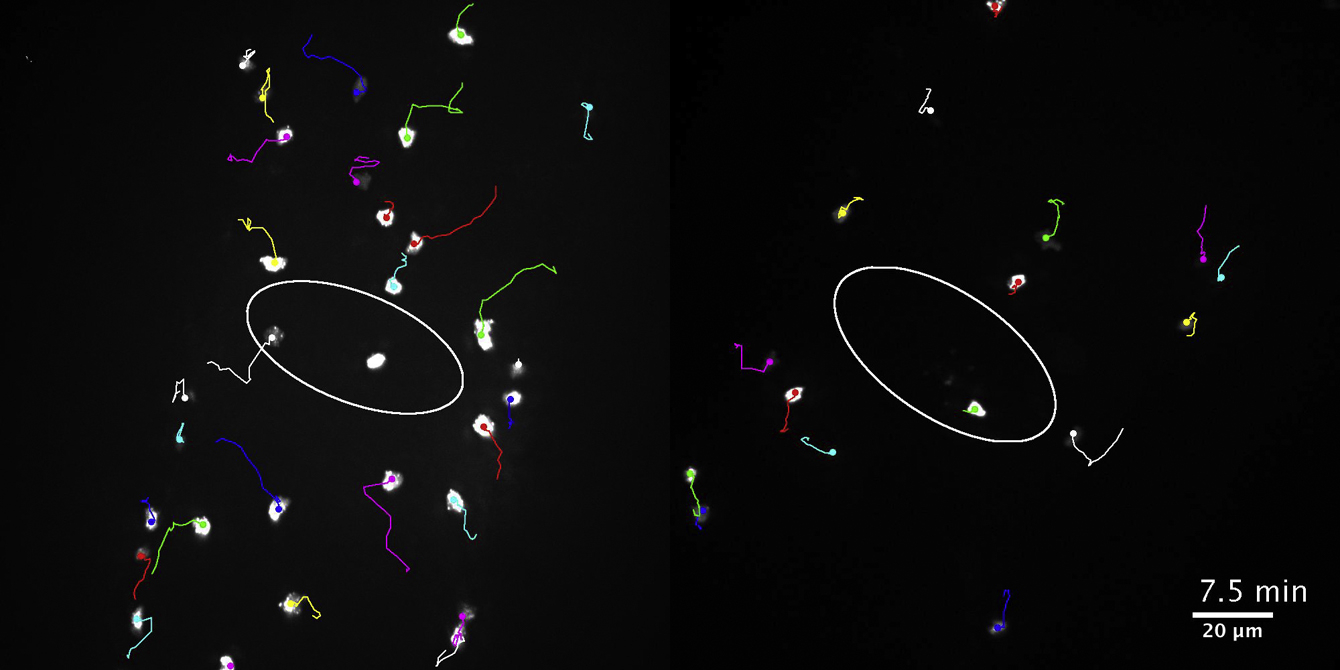

Supplement: Movie S1. Macrophages in src42AE1 Mutants Fail to Respond to Epithelial Wounds, Related to Figure 1 — Twenty-minute movies showing nuclear-red-stinger-labeled macrophages and their tracks in stage 15 embryos immediately after laser wounding in control (left panel) and src42AE1 mutant embryos (right panel). Macrophages fail to respond to the injury appropriately in the src42AE1 mutant, making no net progress towards the wound. Wounds are indicated by white ovals. [file mmc2.jpg]
