# Supplementary material for: Draper/CED-1 Mediates an Ancient Damage Response to Control Inflammatory Blood Cell Migration In Vivo
Source: Curr Biol. 2015 Jun 15;25(12):1606–12. doi: 10.1016/j.cub.2015.04.037 (PMC4503800; doi:10.1016/j.cub.2015.04.037)
Supplement: Document S2. Article plus Supplemental Information [file mmc3.pdf]

Report

Current Biology

Draper/CED-1 Mediates an Ancient Damage Response to Control Inflammatory Blood Cell Migration In Vivo

Highlights

- Draper works downstream of H_2O_2 to drive macrophage migration to wounds in *Drosophila*
- Draper's ITAM domain is critically required for migration of macrophages to wounds
- Draper's ITAM and NPXY motifs have separable functions in *Drosophila* macrophages
- SFK-ITAM-Syk signaling regulates an ancient damage response in *Drosophila*

Authors

Iwan Robert Evans, Frederico S.L.M. Rodrigues, Emma Louise Armitage, Will Wood

Correspondence

i.r.evans@sheffield.ac.uk (I.R.E.),
w.wood@bristol.ac.uk (W.W.)

In Brief

Evans et al. reveal that Draper functions in concert with a Src family kinase (Src42A) and a Syk homolog (Shark) to regulate migration of macrophages to sites of damage in *Drosophila*. This function critically depends upon Draper's immunoreceptor tyrosine-based activation motif and is separable from Draper's canonical role in clearance of dying cells.

Evans et al., 2015, Current Biology *25*, 1606–1612
June 15, 2015 ©2015 The Authors
http://dx.doi.org/10.1016/j.cub.2015.04.037

CellPress

Current Biology
Report

Draper/CED-1 Mediates an Ancient Damage Response to Control Inflammatory Blood Cell Migration In Vivo

Iwan Robert Evans,[1,*] Frederico S.L.M. Rodrigues,[2] Emma Louise Armitage,[1] and Will Wood[2,*]
[1]Department of Infection and Immunity and Bateson Centre, University of Sheffield, Firth Court, Western Bank, Sheffield S10 2TN, UK
[2]School of Cellular and Molecular Medicine, Faculty of Medical Sciences, University of Bristol, Medical Sciences Building, University Walk, Bristol BS8 1TD, UK
*Correspondence: i.r.evans@sheffield.ac.uk (I.R.E.), w.wood@bristol.ac.uk (W.W.)
http://dx.doi.org/10.1016/j.cub.2015.04.037
This is an open access article under the CC BY license (http://creativecommons.org/licenses/by/4.0/).

SUMMARY

Tissue damage leads to a robust and rapid inflammatory response whereby leukocytes are actively drawn toward the wound. Hydrogen peroxide (H_2O_2) has been shown to be an immediate damage signal essential for the recruitment of these inflammatory blood cells to wound sites in both *Drosophila* and vertebrates [1, 2]. Recent studies in zebrafish have shown that wound-induced H_2O_2 is detected by the redox-sensitive Src family kinase (SFK) Lyn within the responding blood cells [3]. Here, we show the same signaling occurs in *Drosophila* inflammatory cells in response to wound-induced H_2O_2 with mutants for the Lyn homolog Src42A displaying impaired inflammatory migration to wounds. We go on to show that activation of Src42A is necessary to trigger a signaling cascade within the inflammatory cells involving the ITAM domain-containing protein Draper-I (a member of the CED-1 family of apoptotic cell clearance receptors) and a downstream kinase, Shark, that is required for migration to wounds. The Src42A-Draper-Shark-mediated signaling axis is homologous to the well-established SFK-ITAM-Syk-signaling pathway used in vertebrate adaptive immune responses. Consequently, our results suggest that adaptive immunoreceptor-signaling pathways important in distinguishing self from non-self appear to have evolved from a more-ancient damage response. Furthermore, this changes the role of H_2O_2 from an inflammatory chemoattractant to an activator signal that primes immune cells to respond to damage cues via the activation of damage receptors such as Draper.

RESULTS AND DISCUSSION

Because H_2O_2 is an evolutionarily conserved, wound-induced damage signal and has been shown to be detected by the redox-sensitive Src family kinase (SFK) Lyn in zebrafish neutrophils [3], we began by investigating whether Lyn's closest relative played a similar role in the recruitment of inflammatory cells to wounds in *Drosophila* embryos. The SFK most closely related to zebrafish Lyn in *Drosophila* is Src42A, and the critical redox-sensitive cysteine residue necessary for H_2O_2 detection in the fish (C466) is conserved in Src42A but is absent from the remaining SFKs and related non-receptor tyrosine kinases (*src62B*, *abl*, and *btk29A*) [3]. Live imaging of macrophage (hemocyte) responses to laser-induced epithelial wounds in *src42A*^{E1} mutant embryos revealed that these inflammatory cells essentially ignored such wounds, exhibiting directionalities close to zero (Figures 1A–1B′; Movie S1). Immunostaining revealed a strong loss of Src activity in these mutant embryos (Figure S1A), and the *src42A*^{E1} wound recruitment defect was phenocopied when placed in a heteroallelic combination with a *src42A*^{myri} loss-of-function allele (Figures S1B′ and S1B″), revealing this inflammatory deficit as specific to *src42A*. Developmental dispersal of macrophages (Figures S1C and S1D) and migration speeds of *src42A* mutant macrophages following injury (Figure 1B″) were indistinguishable from controls, suggesting that their migratory machinery remains intact; therefore defective motility is unlikely to underlie the failure of these cells to respond to wounds. Furthermore, *src42A* does not appear necessary for specification or proliferation of *Drosophila* macrophages, demonstrating a specific role in these cells for *src42A* in wound recruitment (Figures S1C and S1D). In order to test whether the wound recruitment defect observed in *src42A* mutants is due to a macrophage-specific requirement for Src42A, we expressed a dominant-negative version of Src42A (Src42A^{DN}) [4] specifically in macrophages and assessed their ability to respond to wounds. Disrupting Src42A function in this way was sufficient to impair inflammatory recruitment following wounding, demonstrating a cell-autonomous function for Src42A (Figures 1C and 1D).

Expression of Src42A^{DN} within macrophages did not alter overall cellular morphology, though Src42A^{DN} macrophages exhibited slightly larger spread areas in vivo (Figures S1E′ and S1E″) and migrated marginally faster than controls (data not shown). In contrast, macrophages in *src42A* zygotic mutants appeared slightly smaller when visualized live (Figure S1F), potentially reflecting non-macrophage autonomous effects and an altered in vivo environment, which presumably explains the stronger inflammatory defects in these embryos compared to those observed in Src42A^{DN} experiments; for example *src42A* has a role in epidermal responses to injury [5].

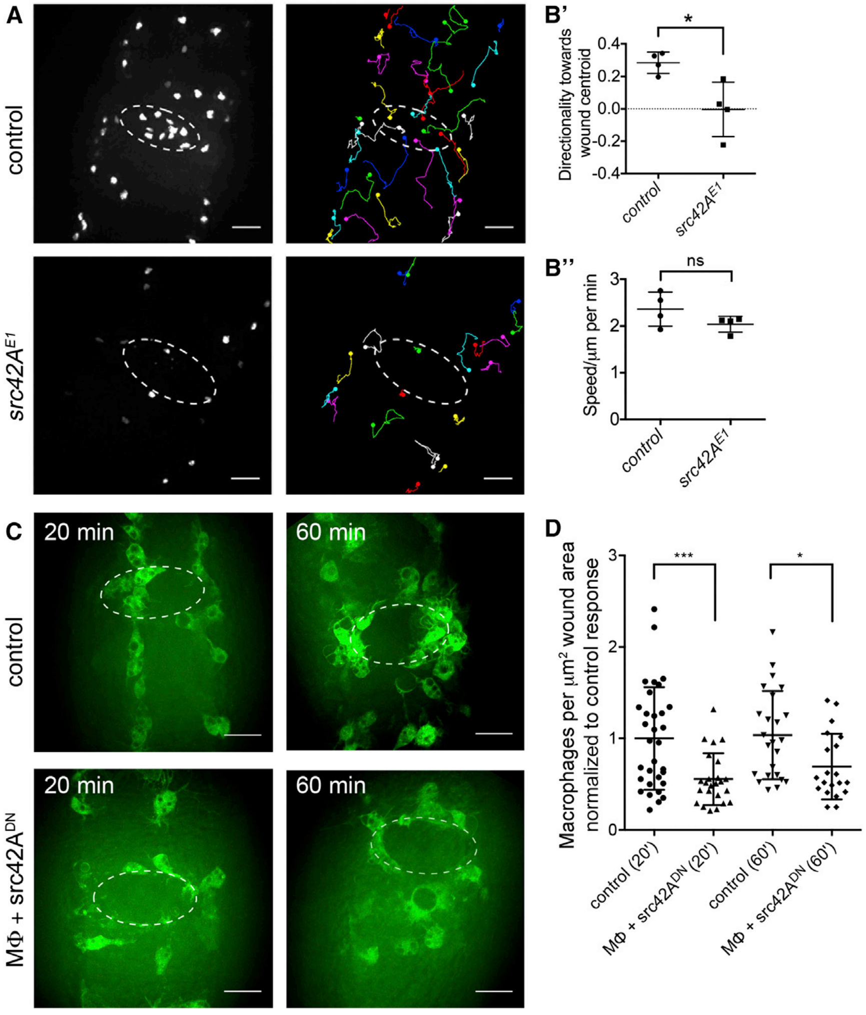

Figure 1. *src42A* Is Required Specifically and Autonomously for Macrophage Wound Responses in *Drosophila* Embryos

(A) Stills and trajectories of red-stinger-labeled macrophages taken at 20 min after wounding from movies of inflammatory responses to wounds in control and $src42A^{E1}$ mutant embryos.

(B) Scatterplots of directionality toward the center of wounds and average speed (per macrophage, per embryo) during wound responses show that macrophages in $src42A^{E1}$ mutants essentially ignore wounds (B′) but that their ability to move at normal speeds is unaffected (B″).

(C) Representative stills of GFP-labeled macrophages (green) at wound sites at 20 and 60 min after wounding in control embryos and embryos expressing a dominant-negative version of Src42A in macrophages.

(D) Scatterplot of wound responses shows numbers of macrophages per μm^2 of wound area normalized according to control averages.

Scale bars represent 20 μm. Central lines and error bars on scatterplots represent mean and SD, respectively; ns, not significant; *$p < 0.05$ and ***$p < 0.001$ via Mann-Whitney test (B) or one-way ANOVA followed by Sidak's multiple comparisons test (D); Mϕ, macrophages; white ovals depict wound edges. See also Figures S1 and S3 and Movie S1.

In summary, *src42A* appears to have a specific role in governing macrophage responses to injury in *Drosophila* embryos and this function is consistent with the related role of zebrafish Lyn in neutrophils [3], where it operates as a redox sensor to alert blood cells to the presence of wound-induced H_2O_2.

What signaling is occurring downstream of Src42A following the detection of H_2O_2? In *Drosophila* glia responding to degenerating axons, *src42A* interacts genetically with the *Drosophila* CED-1 homolog *draper*, where a tyrosine in Draper's ITAM (immunoreceptor tyrosine-based activation motif) domain is critical for these responses [6]. Src42A can phosphorylate Draper at this residue in vitro, and this results in the recruitment of a Syk-related kinase called Shark [6]. In glia, *shark* also genetically interacts with *src42A* and *draper* [6], revealing a tri-partite signaling axis evocative of the well-established SFK-ITAM-Syk-signaling paradigm employed in adaptive immune responses in vertebrates [7]. We therefore wondered whether detection of wound-induced H_2O_2 by Src42A in macrophages might trigger the same signaling pathway and be important for their migration to wounds. Live imaging macrophage responses to wounds in *draper* mutants revealed that these inflammatory migrations are severely impaired with fewer macrophages present at the wound 1 hr after wounding (Figures 2A and 2B). To test whether *draper* was required cell autonomously in macrophages, we used RNAi-mediated knockdown: this approach efficiently depleted *draper-I* levels in macrophages as demonstrated by qPCR for *draper-I* transcripts in FACS-sorted macrophages (Figure S2A), and overexpression of this construct also led to loss of Draper protein in stage 15/16 embryos (Figures S2B′ and S2B″). RNAi-mediated knockdown of *draper* specifically in macrophages led to the same reduction in inflammatory cells at wounds, demonstrating a macrophage-specific requirement for Draper in mediating efficient inflammatory responses to damage (Figures 2A and 2B). We next wanted to determine whether the third participant of the well-established SFK-ITAM-Syk immune signaling pathway was involved in this innate response to damage in vivo: live imaging of macrophage responses to laser wounds in embryos mutant for the Syk homolog *shark* showed that these also have an impaired ability to raise an inflammatory response, with less macrophages arriving at wounds 1 hr post-wounding (Figures 2C, 2D, and S2C). Importantly, we were able to demonstrate specificity and a cell-autonomous role for Shark by re-expression of Shark in macrophages within a $shark^1$ mutant background, an approach that rescues wound responses to control levels (Figures 2E and 2F). A reduction in macrophage numbers does not appear to explain the wound-recruitment phenotypes, because both local and total numbers of macrophages appear unaffected in *draper*, *src42A*, and *shark* mutants (Figures S1C, S1D, and S3). To confirm *src42A* and *draper* operate in the

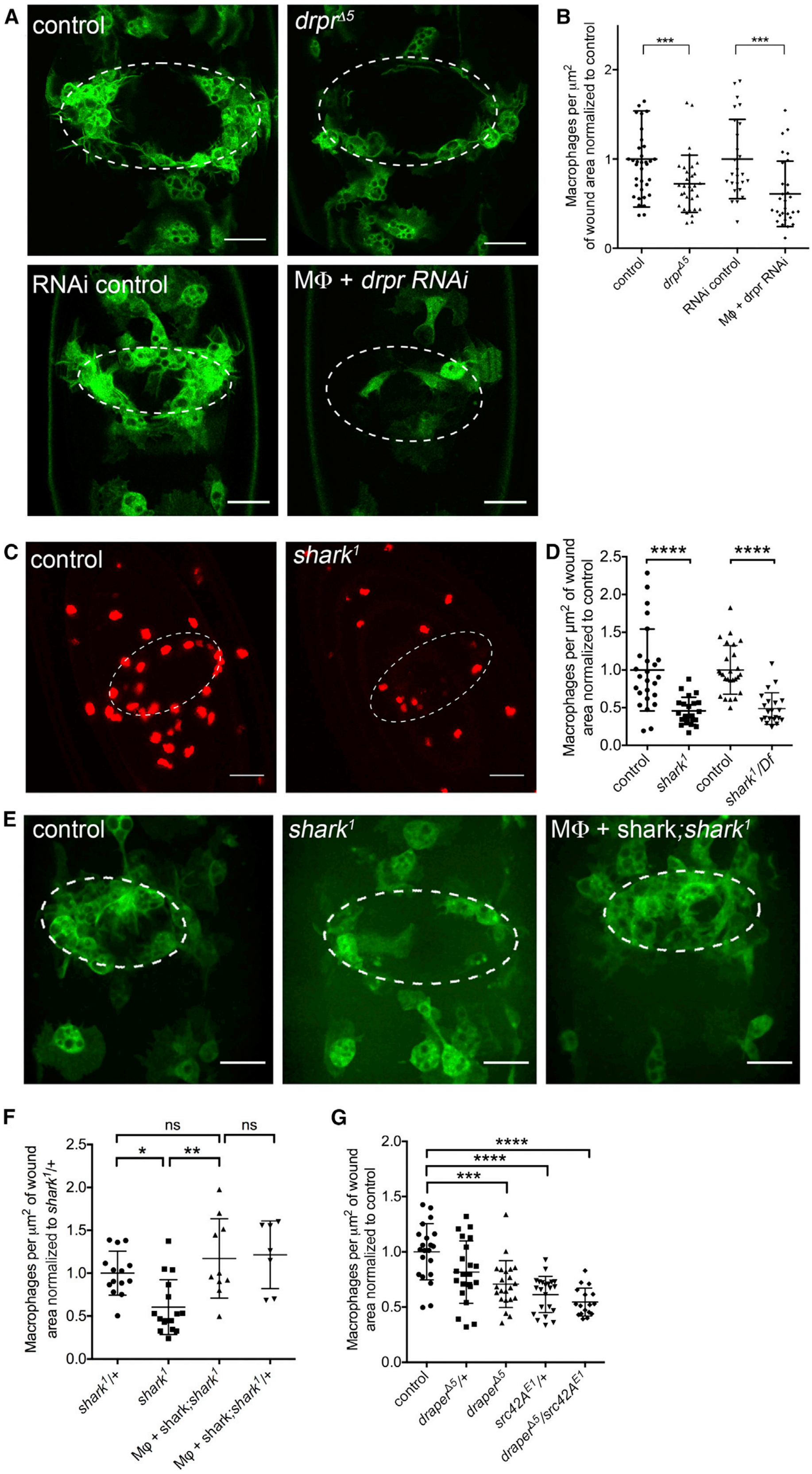

(legend on next page)

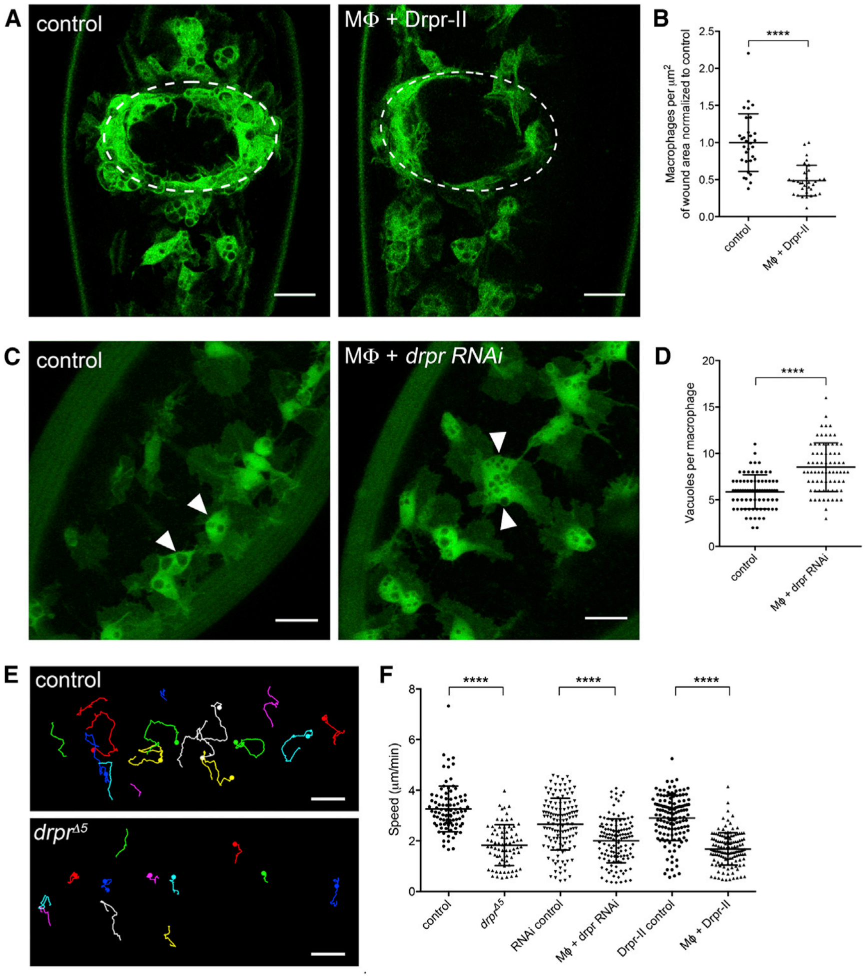

Figure 3. Draper Signaling Is Necessary in Macrophages for Normal Wound Responses, Apoptotic Cell Processing, and Basal Motility

(A) Representative stills of GFP-labeled macrophages (green) at wound sites at 60 min after wounding showing a reduction in immune cell recruitment in embryos in which macrophages express Draper-II, compared to controls; white ovals denote wound outlines.

(B) Scatterplot of wound responses shows defective recruitment of macrophages upon Draper-II expression at 60 min post-wounding.

(C) Stills of GFP-labeled macrophages following macrophage-specific RNAi-mediated knockdown of Draper results in increased vacuolation of these cells, consistent with apoptotic corpse processing defects.

(D) Scatterplot showing increase in number of vacuoles per macrophage on RNAi-mediated knockdown of Draper in macrophages; >30 macrophages from greater than ten embryos analyzed.

(E) Representative macrophage tracks taken from 30-min movies of macrophages migrating in control and *draper* mutant embryos at stage 15, showing a reduction in basal motility in the latter; dots indicate final position of each macrophage—macrophages that leave the plane of focus during the movie terminate without a dot.

(F) Scatterplot of basal motility speeds per macrophage from tracks taken from greater than four movies per genotype. Loss of *draper* function or macrophage-specific expression of an RNAi construct targeting Draper or overexpression of Draper-II reduces the speed of macrophage basal motility at stage 15.

Central lines and error bars on scatterplots represent mean and SD, respectively; ****$p < 0.0001$ via the Mann-Whitney test; scale bars represent 20 μm (A and C) or 25 μm (E). See also Figure S2 and S3.

same genetic pathway, we wounded transheterozygous *src42A/draper* mutant embryos and compared them to controls. The resulting wounds showed a reduction in inflammatory cells present at the wound sites when *src42A*^{E1}/+ heterozygotes were compared with *src42A*^{E1}/*draper*$^{\Delta 5}$ transheterozygotes, suggestive of a genetic interaction (Figure 2G). Taken together, these results suggest that a Src42A-Draper-Shark-signaling axis is critical for the efficient recruitment of inflammatory macrophages to wounds in vivo.

During the late stages of glial responses to axonal injuries, an alternative splice variant, Draper-II, becomes highly upregulated. Rather than an ITAM, the cytoplasmic domain of Draper-II

Figure 2. Cell-Autonomous Requirement for *draper* and *shark* and Genetic Interaction between *src42A* and *draper* during Macrophage Recruitment to Wounds

(A) Representative stills of GFP-labeled macrophages (green) at wound sites at 60 min after wounding in control and *draper*$^{\Delta 5}$ mutant embryos or embryos in which macrophages express a *draper* RNAi construct.

(B) Scatterplot of wound responses shows numbers of macrophages per μm^2 of wound area at 60 min normalized according to control averages.

(C) Representative stills of nuclear-red-stinger-labeled macrophages at 60 min after wounding reveal that embryos with a null mutation in *shark* have a strong reduction in macrophage recruitment to wounds compared to controls.

(D) Scatterplot of wound responses at 60 min reveals defective macrophage recruitment in *shark*^{1} and *shark*^{1}/*Df* embryos.

(E) Representative stills of GFP-labeled macrophages (green) at 60 min post-wounding reveal that overexpression of *shark* in macrophages rescues *shark*^{1} mutant wound response defects.

(F) Scatterplot of wound responses shows rescue of macrophage responses at 60 min with macrophage-specific expression of shark in *shark*^{1} mutant background, compared to *shark*^{1} mutants.

(G) Scatterplot of wound responses in *src42A*^{E1}/*draper* $^{\Delta 5}$ transheterozygotes reveals a genetic interaction of these genes during recruitment of macrophages to wounds.

White ovals display wound margin; central lines and error bars on scatterplots represent mean and SD, respectively; *$p < 0.05$, **$p < 0.01$, ***$p < 0.001$, and ****$p < 0.0001$ according to Mann-Whitney test (B, D, and G) or one-way ANOVA with Sidak's multiple comparisons test (F); scale bars represent 20 μm (A). See also Figure S2 and S3.

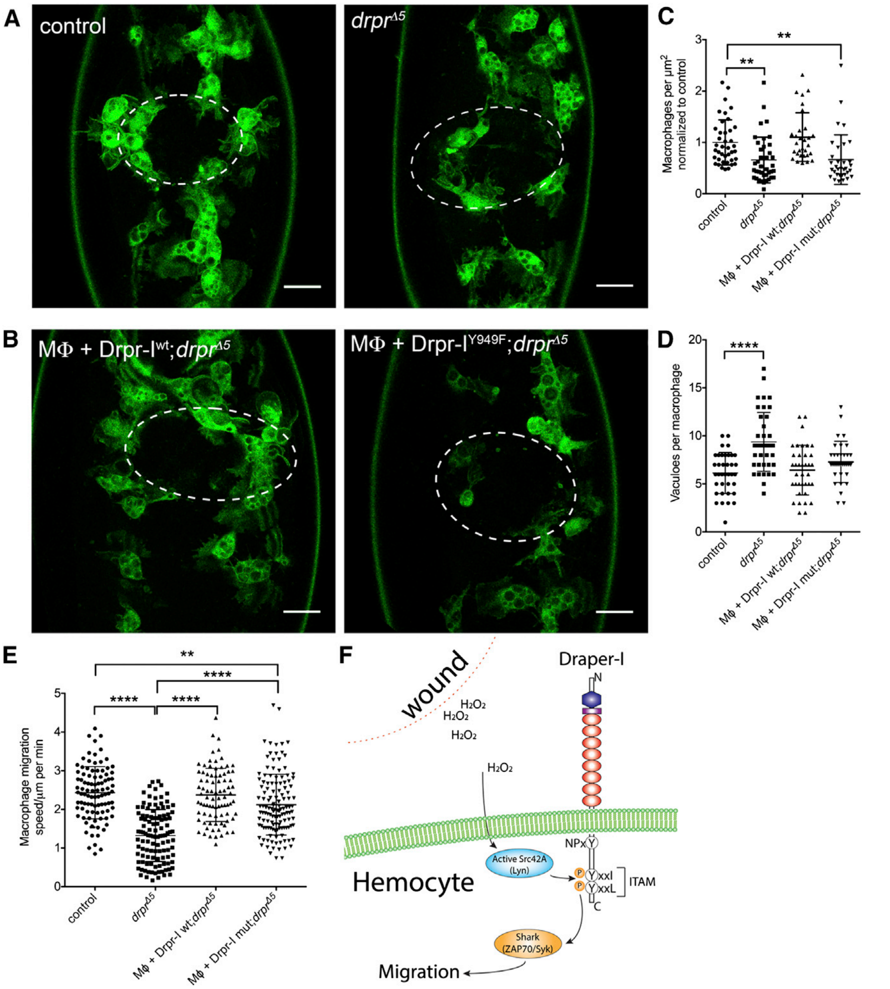

Figure 4. Draper's ITAM Domain Is Specifically Required for Macrophage Chemotactic Migration toward Wounds

(A and B) Representative stills of GFP-labeled macrophages (green) at wound sites at 60 min after wounding in control and *draper* mutant embryos (A) or *draper* mutants with macrophage-specific re-expression of either wild-type Draper-I (MΦ + Drpr-I^{WT};*drpr*$^{\Delta 5}$) or a mutant form of Draper-I lacking a crucial tyrosine residue in its ITAM domain (MΦ + Drpr-I^{Y949F};*drpr*$^{\Delta 5}$; B); white ovals denote wound outlines.

(C) Scatterplots showing rescue of *draper* mutant wound responses on macrophage-specific re-expression of Draper-I^{WT} and a failure of Draper-I^{Y949F} to rescue these responses at 60 min post-wounding.

(D and E) In contrast, the number of vacuoles per macrophage (D; >30 macrophages at the midline analyzed in greater than ten embryos) and the wandering macrophage migration speeds at stage 15 (E; greater than or equal to five movies per genotype) were rescued by expression of either Draper-I^{WT} or Draper-I^{Y949F}.

(F) Schematic illustrating the role of the Src-Draper-Shark-signaling axis in directing inflammatory responses in the *Drosophila* embryo.

Central lines and error bars on scatterplots represent mean and SD, respectively; **p < 0.01 and ****p < 0.0001 according to one-way ANOVA with Sidak's multiple comparisons test; scale bars represent 20 µm (A). See also Figures S3 and S4.

contains an ITIM (immunoreceptor tyrosine inhibitory motif) [8]. Draper-II uses its ITIM to attenuate Draper-I signaling via the recruitment of a phosphatase, Corkscrew, which dephosphorylates Shark [8]. We found that macrophage-specific expression of Draper-II also impaired inflammatory migration to laser-induced epithelial wounds in *Drosophila* embryos (Figures 3A and 3B), further demonstrating a role for Draper signaling in wound responses and highlighting the importance of the ITAM-containing intracellular domain of the Draper-I isoform in this process. ITAMs are found in many mammalian immune receptors involved in adaptive immune responses, such as B cell and T cell receptors, which, as per Draper, can be directly phosphorylated by SFKs [6, 7].

Taken together, these results demonstrate a requirement for SFK-ITAM-Syk signaling in innate immune cell inflammatory responses to damage-induced H_2O_2 and places the ITAM-containing Draper-I variant at the center of this damage-induced signaling cascade. However, *draper* encodes a homolog of the *C. elegans* apoptotic cell clearance receptor CED-1 [9] and has been shown to play a role in the detection and/or the processing of apoptotic debris in both *Drosophila* macrophages and glia [10, 11], the two predominant phagocytes within developing *Drosophila* embryos [12–14]. Indeed, *Drosophila* embryonic macrophages actively prioritize apoptotic cells above the growth factor signals that guide their developmental migrations [2]. A role for the CED-1 family in apoptotic cell clearance appears conserved through to higher vertebrates, because Jedi-1 and MEGF10, the clearest homologs of Draper in mammals, are also involved in the removal of apoptotic cells [15, 16]. Consistent with a role in the efficient processing of engulfed apoptotic debris [11], we found that *draper* mutant and draper RNAi-expressing macrophages appeared vacuolated, containing increased numbers of apoptotic corpses per cell compared to controls (Figures 3C, 3D, and 4D). We have previously shown that efficient processing of engulfed apoptotic corpses is critical for normal macrophage migration to occur [17], and similarly, we were able to observe a reduction in basal migration speeds of macrophages in *draper* mutant embryos or upon macrophage-specific expression of either Draper RNAi or Draper-II (Figures 3E and 3F).

Draper's role in the engulfment and degradation of apoptotic and axonal debris requires the adaptor Ced-6 [18–20], the recruitment of which depends on the presence of an NPXY motif within the cytoplasmic domain of Draper [20, 21]. Our findings, however, suggest that Draper's role in wound detection may be more reliant on its ITAM domain, because despite the presence of an NPXY motif in the Draper-II isoform, expression of Draper-II antagonized wound recruitment. To attempt to

separate Draper's role in clearance and migration, we asked whether expression of a form of Draper-I that lacked the Src phosphorylation site on its ITAM domain (Drpr-I^{Y949F}) [6] was able to rescue the ability of macrophages either to migrate to a wound or process engulfed apoptotic corpses in a *draper*-null mutant background. Crucially, both Draper-I constructs can be expressed at comparable levels, including when expressed in macrophages in a *draper* mutant background (Figures S4A and S4B), while localization and expression levels were very similar in overexpressing macrophages cultured in vitro (Figures S4C′ and S4C″). This suggests there are no intrinsic differences in expression levels as a result of transgene insertion sites, nor are there differences in protein localization between constructs that might undermine this approach. The Y949F mutation strongly perturbs Draper-Shark interactions in vitro [6], and we found that, whereas expression of full-length Draper-I^{WT} in *draper* mutant macrophages was sufficient to rescue their ability to launch an inflammatory response to wounds, expression of the version lacking the Src phosphorylation site was not (Figures 4A–4C). However, the same mutated version of Draper rescued vacuolation defects (Figure 4D) and basal migration rates (Figure 4E) as robustly as the expression of full-length Draper-I^{WT}, demonstrating that, whereas its ITAM domain is critical for Draper's function in macrophage recruitment to wounds, the ITAM domain is dispensable for its role in apoptotic corpse processing, which instead may rely more heavily on the NPXY motif.

Our findings demonstrate a novel role for Draper in the innate immune inflammatory response to wounds and place it downstream of the early damage cue H_2O_2. Our results suggest that H_2O_2 production at wounds is detected by Src42A acting as a redox sensor within macrophages and that this then triggers the phosphorylation of Draper-I on its ITAM domain and the downstream recruitment and activation of the kinase Shark (Figure 4F). SFK-ITAM-Syk signaling is a well-established immune-signaling pathway used in the mammalian adaptive immune response during B cell and T cell signaling.

Our results suggest that this adaptive immune-signaling pathway important in distinguishing self from non-self appears to have evolved from a more-ancient damage response and changes the role of H_2O_2 from an inflammatory chemoattractant to an activator signal that potentially primes immune cells to respond to damage cues via the activation of damage receptors such as Draper and possibly other ITAM domain-containing proteins.

EXPERIMENTAL PROCEDURES

Drosophila Stocks and Genetics

For a full list of genotypes used in this study, see Table S1. Crosses were performed in laying cages with apple juice agar plates and flies left to lay overnight at 22°C with embryos collected the following day, with the exception of the Src42A^{DN} and *draper* RNAi overexpression experiments, in which embryos were laid at 29°C overnight. *Drosophila* mutants and transgenic lines were obtained from the Bloomington Stock Centre unless otherwise stated (see Table S2 for details). Further information on the lines used can also be found in Flybase [22]. Inserts of *srp-GAL4* [23], *crq-GAL4*, and *pxn-GAL4* [24] were recombined with either *UAS-EGFP* or *UAS-nuclear red stinger* in order to track macrophage movement and morphology, whereas *da-GAL4* [25] was used to drive ubiquitous expression from UAS transgenes. The following mutants and transgenes were used in this study: w^{1118} (as a "wild-type" control background), $src42A^{E1}$ [26], $src42A^{K10108}$ [27, 28], $src42A^{myri}$ [29], $shark^{1}$ [30], the *shark*-deleting deficiency *Df(2L)BSC434* [31], $draper^{\Delta5}$ [9], *UAS-draper RNAi*^{HM501623} [32], *UAS-src42A*^{DN} [4], *UAS-draper-I*^{WT} [33], *UAS-shark-2* [30], *UAS-draper-I*^{Y949F} line 24127-5 [8], and *UAS-draper-II* line 03529-6 [8]. Mutants were discriminated through selection against *CTG*, *TTG*, or *GAL4*-independent fluorescent balancer chromosomes [34, 35].

Live Imaging of *Drosophila* Macrophages

For all live imaging experiments, stage 15 embryos were collected from overnight apple juice agar plates and mounted on slides in a minimal volume of 10S Voltalef oil (VWR), following dechorionation in bleach for 1 min and extensive washing in water. All imaging was carried out at room temperature. For dynamic imaging of wound responses, epithelial wounds were induced using a nitrogen-pumped Micropoint ablation laser tuned to 435 nm (Andor Technologies), as per Razzell et al. [36]. EGFP or nuclear-red-stinger-expressing macrophages were followed at 30-s intervals for 20 min post-wounding using a 40× oil immersion objective lens on a PerkinElmer UltraView spinning disc system. Basal migration speeds were analyzed by making time-lapse movies of macrophages specifically expressing either nuclear red stinger or EFGP in embryos on the ventral side of the embryo at stage 15 of development using a Leica LSM510 confocal and a 40× oil immersion objective. Movies were as per Evans et al. [17], with z stacks collected every 30 s for 20 min. Wound responses were also quantified at 20 and 60 min post-wounding (numbers of macrophages per μm^2 of wound, normalized to the average wound response of the control). For a detailed description of image processing and analysis, see the Supplemental Experimental Procedures.

SUPPLEMENTAL INFORMATION

Supplemental Information includes Supplemental Experimental Procedures, four figures, two tables, and one movie and can be found with this article online at http://dx.doi.org/10.1016/j.cub.2015.04.037.

AUTHOR CONTRIBUTIONS

I.R.E., F.S.L.M.R., and W.W. conceived and designed the experiments; I.R.E., F.S.L.M.R., and E.L.A. performed the experiments; and I.R.E., F.S.L.M.R., E.L.A., and W.W. wrote the paper.

ACKNOWLEDGMENTS

We are particularly indebted to Poonam Ghai, Kate Comber, and Isabella Vlisidou for technical assistance and Marc Freeman (UMASS) and Mary Logan (OHSU) for advice and reagents. The authors wish to acknowledge the assistance of Adrian Rogers (University of Bath Imaging Facility) and Darren Robinson (University of Sheffield Light Microscopy Facility) for imaging and of Andrew Herman (University of Bristol Faculty of Medical and Veterinary Sciences Flow Cytometry Facility) for cell sorting. This work would be impossible without stocks from the Bloomington *Drosophila* Stock Centre (NIH P40OD018537) and use of Flybase. We thank the TRiP at Harvard Medical School (NIH/NIGMS R01-GM084947) for providing transgenic RNAi fly stocks used in this study and also Paul Martin (University of Bristol), Brian Stramer (KCL), Eric Baehrecke (UMASS), and David Strutt (University of Sheffield) for sharing fly lines. W.W. is a Wellcome Trust Senior Fellow (grant 090899/Z/09/Z); I.R.E. is a Wellcome Trust/Royal Society Sir Henry Dale Fellow (grant 102503/Z/13/Z). This work was also funded by a Thomas-Berry and Simpson Fellowship awarded to I.R.E. by the University of Sheffield.

Received: October 21, 2014
Revised: March 16, 2015
Accepted: April 16, 2015
Published: May 28, 2015

REFERENCES

1. Niethammer, P., Grabher, C., Look, A.T., and Mitchison, T.J. (2009). A tissue-scale gradient of hydrogen peroxide mediates rapid wound detection in zebrafish. Nature *459*, 996–999.

2. Moreira, S., Stramer, B., Evans, I., Wood, W., and Martin, P. (2010). Prioritization of competing damage and developmental signals by migrating macrophages in the Drosophila embryo. Curr. Biol. *20*, 464–470.
3. Yoo, S.K., Starnes, T.W., Deng, Q., and Huttenlocher, A. (2011). Lyn is a redox sensor that mediates leukocyte wound attraction in vivo. Nature *480*, 109–112.
4. Shindo, M., Wada, H., Kaido, M., Tateno, M., Aigaki, T., Tsuda, L., and Hayashi, S. (2008). Dual function of Src in the maintenance of adherens junctions during tracheal epithelial morphogenesis. Development *135*, 1355–1364.
5. Juarez, M.T., Patterson, R.A., Sandoval-Guillen, E., and McGinnis, W. (2011). Duox, Flotillin-2, and Src42A are required to activate or delimit the spread of the transcriptional response to epidermal wounds in Drosophila. PLoS Genet. *7*, e1002424.
6. Ziegenfuss, J.S., Biswas, R., Avery, M.A., Hong, K., Sheehan, A.E., Yeung, Y.G., Stanley, E.R., and Freeman, M.R. (2008). Draper-dependent glial phagocytic activity is mediated by Src and Syk family kinase signalling. Nature *453*, 935–939.
7. Underhill, D.M., and Goodridge, H.S. (2007). The many faces of ITAMs. Trends Immunol. *28*, 66–73.
8. Logan, M.A., Hackett, R., Doherty, J., Sheehan, A., Speese, S.D., and Freeman, M.R. (2012). Negative regulation of glial engulfment activity by Draper terminates glial responses to axon injury. Nat. Neurosci. *15*, 722–730.
9. Freeman, M.R., Delrow, J., Kim, J., Johnson, E., and Doe, C.Q. (2003). Unwrapping glial biology: Gcm target genes regulating glial development, diversification, and function. Neuron *38*, 567–580.
10. Manaka, J., Kuraishi, T., Shiratsuchi, A., Nakai, Y., Higashida, H., Henson, P., and Nakanishi, Y. (2004). Draper-mediated and phosphatidylserine-independent phagocytosis of apoptotic cells by Drosophila hemocytes/macrophages. J. Biol. Chem. *279*, 48466–48476.
11. Kurant, E., Axelrod, S., Leaman, D., and Gaul, U. (2008). Six-microns-under acts upstream of Draper in the glial phagocytosis of apoptotic neurons. Cell *133*, 498–509.
12. Abrams, J.M., White, K., Fessler, L.I., and Steller, H. (1993). Programmed cell death during Drosophila embryogenesis. Development *117*, 29–43.
13. Tepass, U., Fessler, L.I., Aziz, A., and Hartenstein, V. (1994). Embryonic origin of hemocytes and their relationship to cell death in Drosophila. Development *120*, 1829–1837.
14. Kurant, E. (2011). Keeping the CNS clear: glial phagocytic functions in Drosophila. Glia *59*, 1304–1311.
15. Wu, H.H., Bellmunt, E., Scheib, J.L., Venegas, V., Burkert, C., Reichardt, L.F., Zhou, Z., Fariñas, I., and Carter, B.D. (2009). Glial precursors clear sensory neuron corpses during development via Jedi-1, an engulfment receptor. Nat. Neurosci. *12*, 1534–1541.
16. Scheib, J.L., Sullivan, C.S., and Carter, B.D. (2012). Jedi-1 and MEGF10 signal engulfment of apoptotic neurons through the tyrosine kinase Syk. J. Neurosci. *32*, 13022–13031.
17. Evans, I.R., Ghai, P.A., Urbančič, V., Tan, K.L., and Wood, W. (2013). SCAR/WAVE-mediated processing of engulfed apoptotic corpses is essential for effective macrophage migration in Drosophila. Cell Death Differ. *20*, 709–720.
18. Awasaki, T., Tatsumi, R., Takahashi, K., Arai, K., Nakanishi, Y., Ueda, R., and Ito, K. (2006). Essential role of the apoptotic cell engulfment genes draper and ced-6 in programmed axon pruning during Drosophila metamorphosis. Neuron *50*, 855–867.
19. Cuttell, L., Vaughan, A., Silva, E., Escaron, C.J., Lavine, M., Van Goethem, E., Eid, J.P., Quirin, M., and Franc, N.C. (2008). Undertaker, a Drosophila Junctophilin, links Draper-mediated phagocytosis and calcium homeostasis. Cell *135*, 524–534.
20. Kuraishi, T., Nakagawa, Y., Nagaosa, K., Hashimoto, Y., Ishimoto, T., Moki, T., Fujita, Y., Nakayama, H., Dohmae, N., Shiratsuchi, A., et al. (2009). Pretaporter, a Drosophila protein serving as a ligand for Draper in the phagocytosis of apoptotic cells. EMBO J. *28*, 3868–3878.
21. Fujita, Y., Nagaosa, K., Shiratsuchi, A., and Nakanishi, Y. (2012). Role of NPxY motif in Draper-mediated apoptotic cell clearance in Drosophila. Drug Discov. Ther. *6*, 291–297.
22. St Pierre, S.E., Ponting, L., Stefancsik, R., and McQuilton, P.; FlyBase Consortium (2014). FlyBase 102—advanced approaches to interrogating FlyBase. Nucleic Acids Res. *42*, D780–D788.
23. Brückner, K., Kockel, L., Duchek, P., Luque, C.M., Rørth, P., and Perrimon, N. (2004). The PDGF/VEGF receptor controls blood cell survival in Drosophila. Dev. Cell *7*, 73–84.
24. Stramer, B., Wood, W., Galko, M.J., Redd, M.J., Jacinto, A., Parkhurst, S.M., and Martin, P. (2005). Live imaging of wound inflammation in Drosophila embryos reveals key roles for small GTPases during in vivo cell migration. J. Cell Biol. *168*, 567–573.
25. Wodarz, A., Hinz, U., Engelbert, M., and Knust, E. (1995). Expression of crumbs confers apical character on plasma membrane domains of ectodermal epithelia of Drosophila. Cell *82*, 67–76.
26. Tateno, M., Nishida, Y., and Adachi-Yamada, T. (2000). Regulation of JNK by Src during Drosophila development. Science *287*, 324–327.
27. Lu, X., and Li, Y. (1999). Drosophila Src42A is a negative regulator of RTK signaling. Dev. Biol. *208*, 233–243.
28. Spradling, A.C., Stern, D., Beaton, A., Rhem, E.J., Laverty, T., Mozden, N., Misra, S., and Rubin, G.M. (1999). The Berkeley Drosophila Genome Project gene disruption project: single P-element insertions mutating 25% of vital Drosophila genes. Genetics *153*, 135–177.
29. Takahashi, F., Endo, S., Kojima, T., and Saigo, K. (1996). Regulation of cell-cell contacts in developing Drosophila eyes by Dsrc41, a new, close relative of vertebrate c-src. Genes Dev. *10*, 1645–1656.
30. Fernandez, R., Takahashi, F., Liu, Z., Steward, R., Stein, D., and Stanley, E.R. (2000). The Drosophila shark tyrosine kinase is required for embryonic dorsal closure. Genes Dev. *14*, 604–614.
31. Cook, R.K., Christensen, S.J., Deal, J.A., Coburn, R.A., Deal, M.E., Gresens, J.M., Kaufman, T.C., and Cook, K.R. (2012). The generation of chromosomal deletions to provide extensive coverage and subdivision of the Drosophila melanogaster genome. Genome Biol. *13*, R21.
32. Dietzl, G., Chen, D., Schnorrer, F., Su, K.C., Barinova, Y., Fellner, M., Gasser, B., Kinsey, K., Oppel, S., Scheiblauer, S., et al. (2007). A genome-wide transgenic RNAi library for conditional gene inactivation in Drosophila. Nature *448*, 151–156.
33. McPhee, C.K., Logan, M.A., Freeman, M.R., and Baehrecke, E.H. (2010). Activation of autophagy during cell death requires the engulfment receptor Draper. Nature *465*, 1093–1096.
34. Halfon, M.S., Gisselbrecht, S., Lu, J., Estrada, B., Keshishian, H., and Michelson, A.M. (2002). New fluorescent protein reporters for use with the Drosophila Gal4 expression system and for vital detection of balancer chromosomes. Genesis *34*, 135–138.
35. Le, T., Liang, Z., Patel, H., Yu, M.H., Sivasubramaniam, G., Slovitt, M., Tanentzapf, G., Mohanty, N., Paul, S.M., Wu, V.M., and Beitel, G.J. (2006). A new family of Drosophila balancer chromosomes with a w-dfd-GMR yellow fluorescent protein marker. Genetics *174*, 2255–2257.
36. Razzell, W., Evans, I.R., Martin, P., and Wood, W. (2013). Calcium flashes orchestrate the wound inflammatory response through DUOX activation and hydrogen peroxide release. Curr. Biol. *23*, 424–429.

Current Biology

Supplemental Information

Draper/CED-1 Mediates an Ancient Damage Response to Control Inflammatory Blood Cell Migration In Vivo

Iwan Robert Evans, Frederico S.L.M. Rodrigues, Emma Louise Armitage, and Will Wood

Supplemental Data

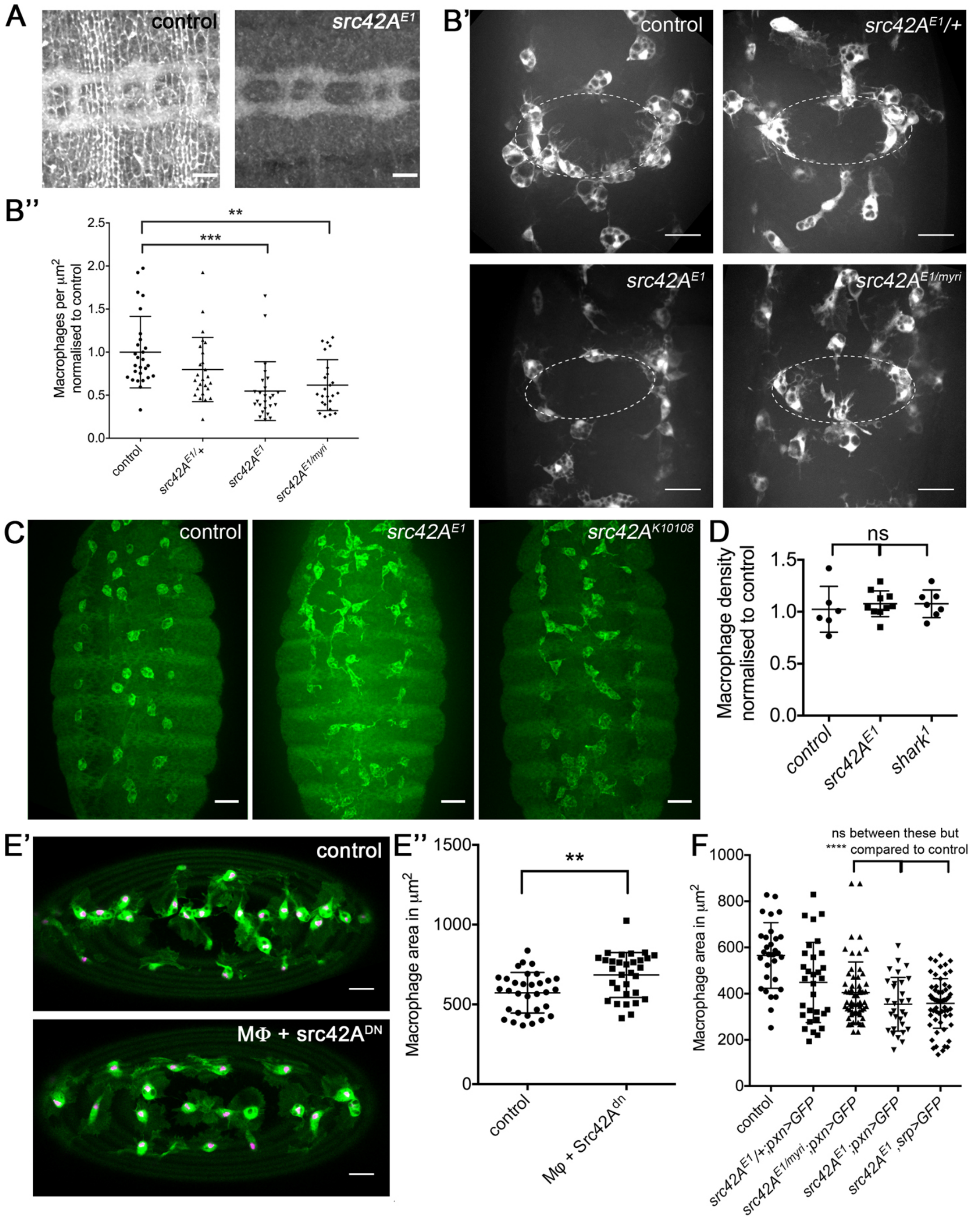

Figure S1, relates to Figure 1. *src42A* heteroallelic combinations recapitulate *src42A*^{E1} wound defects but macrophage developmental dispersal and specification appears unaffected in *src42A* mutants

To confirm loss of *src42A* function, control and *src42A*^{E1} embryos were immunostained for pSrc, showing a significant reduction in overall levels of active Src (A). Representative stills of *pxn-Gal4,UAS-GFP*-labelled macrophages at wound sites 60 min post-wounding in control,

src42A^{E1}/+ heterozygous, *src42A*^{E1} homozygous and *src42A*$^{E1/myri}$ heteroallelic embryos (B'); white ovals denote wounds. Scatterplot of wound responses reveal that heteroallelic combinations of *src42A* recapitulate *src42A*^{E1} mutant defects in wound recruitment at 60 min post-wounding (B''). Representative images of the ventral side of stage 15 embryos immunostained for Fascin (green) demonstrate that specification and developmental migration of macrophages appear normal in *src42A* mutants (C). Expression using *pxn-Gal4* as an alternative macrophage marker also appeared normal and this was used to assess local macrophage density on the ventral midline at stage 15 in control, *src42A*^{E1} and *shark*^{1} mutant embryos (D). Normal migration and marker expression suggests macrophages are correctly specified and carry out their other functions normally in the absence of *src42A*. Similarly, macrophages expressing GFP (green) and red stinger (purple) that also express Src42A^{DN} disperse and appear morphologically normal (E'), but do exhibit a small but statistically significant increase in spread area (E''). In contrast, increasingly potent combinations of *src42A* loss-of-function alleles correlated with a reduction in spread area (F). Scale bars represent 10 µm (A) or 20 µm (B'-C and E'); central lines and error bars on scatterplots represent mean and standard deviation, respectively; **, *** and **** denote $p<0.01$, $p<0.001$ and 0.0001, respectively; via one-way ANOVA with Sidak's multiple comparisons test (B'', D and F) or the Mann-Whitney test (E''); Mϕ = macrophages.

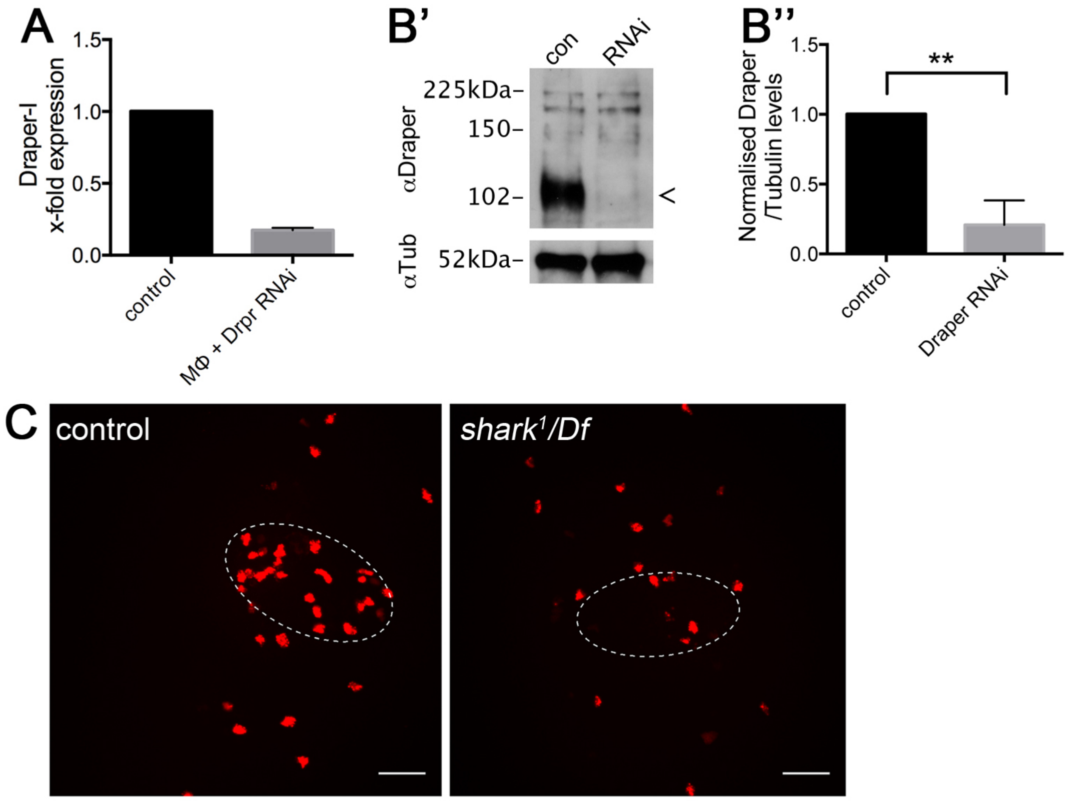

Figure S2, relates to Figures 2 and 3. Controls showing efficient knockdown of Draper via RNAi and heteroallelic *shark* loss-of-function wound images

Real-time PCR analysis of the relative expression levels of *draper-I* in embryonic stage 15 sorted macrophages was carried out following RNAi-mediated knockdown of *draper*. Draper threshold cycle (Ct) mRNA values were normalised to ribosomal protein L32 and results are presented as fold-reduction relative to control (A). Representative western blot of Draper levels in stage 15/16 embryos following *da-Gal4*-driven ubiquitous expression of the same *draper* RNAi construct; arrowhead indicates position of Draper protein (B'). Quantification of Draper knockdown, normalised to the αTubulin loading control and shown in relation to Draper-I^{wt} levels (B''; 3 biological replicates, $p<0.01$ via Student's T-test). To confirm *shark* phenotypes were not the result of a mutation elsewhere on the *shark^{1}*-bearing chromosome, wound recruitment was probed in embryos containing heteroallelic *shark* loss-of-function mutations. Representative images of control and *shark^{1}/Df* embryos showing defective recruitment of red stinger-labelled macrophages to wounds at 60 min post-wounding (C); white ovals denote wounds. N.b. quantification of this data is shown in Figure 2D. Graphs show mean and standard deviation; scale bars represent 20 μm.

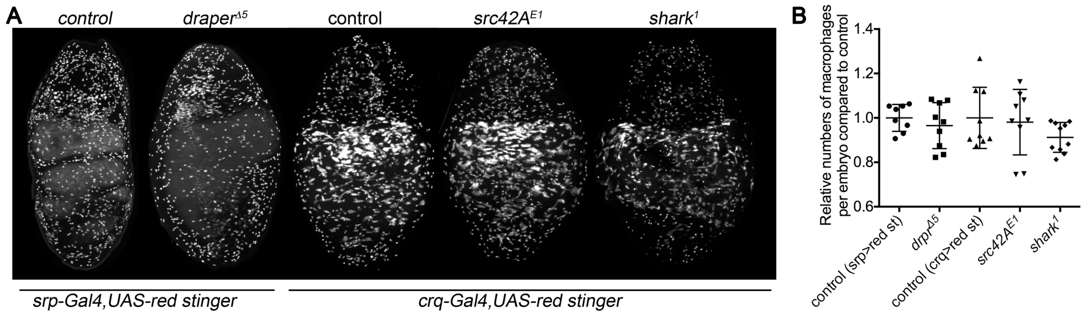

Figure S3, relates to all Figures. No differences in overall numbers of embryonic macrophages per embryo in the mutants used in this study

Representative images of squashed fly st15/16 embryos with red stinger-expressing macrophages (A). Scatterplot showing numbers of macrophages per embryo compared to controls (B); no statistically significant differences in overall numbers of macrophages was found between mutants and controls using Student's T-test (control vs *draper*$^{\Delta 5}$) or a one-way ANOVA with Sidak's multiple comparisons test (control, *src42A*^{E1} and *shark*^{1}); central and outer bars represent mean and standard deviation, respectively.

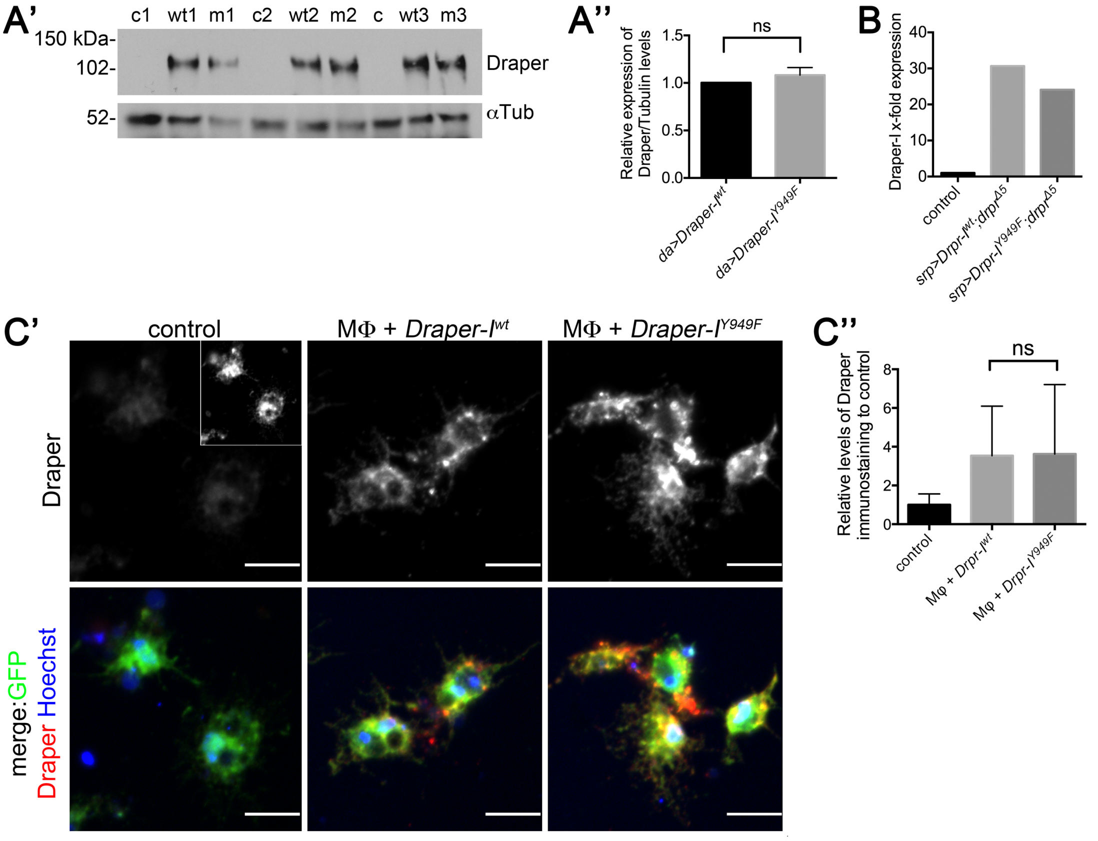

Figure S4, relates to Figure 4. Draper-I^{wt} and Draper-I^{Y949F} localise and are expressed at similar levels to each other

In order to verify comparable expression of Draper-I^{wt} (wt) and Draper-I^{Y949F} (m) protein, stage 15/16 embryos ± overexpression of each construct (via *da-Gal4*) were analysed by western blotting (A’; c = control). No difference was found between expression levels of the wild type or mutant Draper construct (A’’; n=3 biological replicates with 3 blots per replicate). Real-time PCR analysis of relative expression levels of *draper-I* mRNA in whole embryos following following *srp-Gal4*-mediated *drpr-I^{wt} and drpr-I^{Y949F}* overexpression in macrophages in a *drpr^{Δ5}* mutant background. Draper threshold cycle (Ct) mRNA values were normalised to ribosomal protein L32 and results are presented as fold-reduction relative to control (B). GFP-labelled macrophages ± *srp-Gal4;crq-Gal4*-mediated expression of each construct were cultured in vitro and immunostained for Draper and GFP (C’) revealing a similar subcellular localisation and similar expression levels; inset shows contrast enhanced control macrophages demonstrating a related localisation pattern for endogenous Draper. Quantification of anti-Draper immunofluorescence intensities per cell also indicated comparable expression levels for each construct (C’’), though both represented a significant increase compared to

endogenous levels (>35 cells per genotype quantified from 3 biological replicates). Mϕ = macrophages; scale bars represent 10 µm; ns denotes lack of statistical significance, Student's T-test (A) or one-way ANOVA with Sidak's multiple comparisons test (C''). Bar charts show mean; error bars represent the standard deviation (A", B, C").

Supplemental Tables

Table S1, relates to experimental procedures. Genotypes of embryos used:

Figure:	Label:	Genotype:
**Fig. 1A, B**	control	*w;;crq-GAL4,UAS-nuclear red stinger*
	src42A^{E1}	*w;src42E1;crq-GAL4,UAS-nuclear red stinger*
**Fig. 1C, D**	control	*w;srp-GAL4,UAS-EGFP/+;crq-GAL4,UAS-EGFP/+*
	macrophages + src42A^{DN}	*w;srp-GAL4,UAS-gfp/+;crq-GAL4,UAS-gfp/UAS-src42A*^{DN}
**Fig. 2A, B**	control	*w;srp-GAL4,UAS-EGFP*
	draper$^{\Delta5}$	*w;srp-GAL4,UAS-EGFP;draper*$^{\Delta5}$
	RNAi control	*;srp-GAL4,UAS-GFP/+;crq-GAL4,UAS-EGFP/+*
	macrophages + *drpr RNAi*	*;srp-GAL4,UAS-GFP/UAS-draper RNAi HM501623;crq-GAL4,UAS-EGFP/+*
**Fig. 2C, D**	control	*w;;crq-GAL4,UAS-nuclear red stinger*
	shark^{1}	*w;shark*^{1}*;crq-GAL4,UAS-nuclear red stinger*
**Fig. 2D**	control	*w;;crq-GAL4,UAS-nuclear red stinger*
	shark^{1}*/Df*	*w;shark*^{1}*/Df(2L)BSC434;crq-GAL4,UAS-nuclear red stinger*
**Fig. 2E, F**	*shark*^{1}*/+*	*w;shark*^{1}*,srp-Gal4,UAS-EGFP/+*
	shark^{1}	*w;shark*^{1}*,srp-Gal4,UAS-EGFP/shark*^{1}
	macrophages + shark*;shark*^{1}	*w,UAS-shark-2/w or Y;shark*^{1}*,srp-GAL4,UAS-EGFP/shark*^{1}
	macrophages + shark*;shark*^{1}*/+*	*w,UAS-shark-2/w or Y;shark*^{1}*,srp-GAL4,UAS-EGFP/CyO ro-tLacZ*
**Fig. 2G**	control	*w;srp-GAL4,UAS-EGFP/srp-GAL4,UAS-nuclear red stinger*
	draper$^{\Delta5}$*/+*	*w;srp-GAL4,UAS-EGFP/srp-GAL4,UAS-nuclear red stinger;draper*$^{\Delta5}$*/+*
	draper$^{\Delta5}$	*w;srp-GAL4,UAS-EGFP/srp-GAL4,UAS-nuclear red stinger;draper*$^{\Delta5}$
	src42A^{E1}*/+*	*w;src42*^{E1}*/srp-Gal4,UAS-EGFP;crq-GAL4,UAS-nuclear red stinger/+*
	draper$^{\Delta5}$*/src42A*^{E1}	*w;src42*^{E1}*/srp-Gal4,UAS-EGFP;crq-GAL4,UAS-nuclear red stinger/draper*$^{\Delta5}$
**Fig. 3A, B**	control	*w;srp-GAL4,UAS-EGFP/+;crq-GAL4,UAS-EGFP/+*
	macrophages + Drpr-II	*w;srp-GAL4,UAS-GFP/UAS-draper-II;crq-GAL4,UAS-EGFP/+*
**Fig. 3C, D**	control	*w;srp-GAL4,UAS-GFP/+;crq-GAL4,UAS-EGFP/+*
	macrophages + *drpr RNAi*	*;srp-GAL4,UAS-GFP/UAS-draper RNAi HM501623;crq-GAL4,UAS-EGFP/+*
**Fig. 3E**	control	*w;srp-GAL4,UAS-nuclear red stinger*
	draper$^{\Delta5}$	*w;srp-GAL4,UAS-nuclear red stinger;draper*$^{\Delta5}$
**Fig. 3F**	control	*w;srp-Gal4,UAS-nuclear red stinger*
	draper$^{\Delta5}$	*w;srp-GAL4,UAS-nuclear red stinger;draper*$^{\Delta5}$
	RNAi control	*w;srp-GAL4,UAS-GFP/+;crq-GAL4,UAS-EGFP/+*
	macrophages + *drpr RNAi*	*;srp-GAL4,UAS-GFP/UAS-draper RNAi HM501623;crq-GAL4,UAS-EGFP/+*
	Drpr-II control	*w;srp-GAL4,UAS-EGFP/+;crq-GAL4,UAS-EGFP/+*
	macrophages + Drpr-II	*w;srp-GAL4,UAS-GFP/UAS-draper-II;crq-GAL4,UAS-EGFP/+*
**Fig. 4**	control	*w;srp-GAL4,UAS-EGFP/srp-GAL4,UAS-nuclear red stinger*
	draper$^{\Delta5}$	*w;srp-GAL4,UAS-EGFP/srp-GAL4,UAS-nuclear red stinger;draper*$^{\Delta5}$
	macrophages + Drpr-I^{wt}*;draper*$^{\Delta5}$	*w;srp-GAL4,UAS-GFP/srp-GAL4,UAS-draper-I*^{wt}*;draper*$^{\Delta5}$
	macrophages + Drpr-I^{Y949F}*;draper*$^{\Delta5}$	*w;srp-GAL4,UAS-GFP/srp-GAL4,UAS-draper-I*^{Y949F}*;draper*$^{\Delta5}$

Figure:	Label:	Genotype:
**Supplementary Fig. 1A**	control	*w*
	src42^{E1}	*w;src42A*^{E1}

Table S1 continued:

**Supplementary Fig. 1B', B''**	control	*w;;pxn-GAL4,UAS-EGFP*
	src42A^{E1}/+	*w;src42A*$^{E1/+}$*;pxn-GAL4,UAS-EGFP*
	src42^{E1}	*w;src42A*^{E1}*;pxn-GAL4,UAS-EGFP*
	src42$^{E1/myri}$	*w;src42A*^{E1}*/src42A*^{myri}*;pxn-Gal4,UAS-EGFP*
**Supplementary Fig. 1C**	control	*w*
	src42^{E1}	*w;src42A*^{E1}
	src42A^{K10108}	*w;src42A*^{K10108}
**Supplementary Fig. 1D**	control	*w;;pxn-GAL4,UAS-EGFP*
	src42^{E1}	*w;src42A*^{E1}*;pxn-GAL4,UAS-EGFP*
	shark^{1}	*w;shark*^{1}*;pxn-GAL4,UAS-EGFP*
**Supplementary Fig. 1E', E''**	control	*w;srp-GAL4,UAS-EGFP/+;crq-GAL4,UAS-nuclear red stinger/+*
	macrophages + src42A^{DN}	*w;srp-GAL4,UAS-EGFP/+;crq-GAL4,UAS-nuclear red stinger/UAS-src42A*^{DN}
**Supplementary Fig. 1F**	control	*w;;pxn-GAL4,UAS-EGFP*
	src42A^{E1}*/+;pxn>GFP*	*w;src42A*^{E1}*/+;pxn-GAL4,UAS-EGFP*
	src42A$^{E1/myri}$*;pxn>GFP*	*w;src42A*^{E1}*/src42A*^{myri}*;pxn-Gal4,UAS-EGFP*
	src42A^{E1}*;pxn>GFP*	*w;src42A*^{E1}*;pxn-GAL4,UAS-EGFP*
	src42A^{E1}*,srp>GFP*	*w;src42A*^{E1}*,srp-GAL4,UAS-EGFP*
**Supplementary Fig. 2A**	control	*w;srp-GAL4,UAS-GFP/+;crq-GAL4,UAS-EGFP/+*
	macrophages + Draper RNAi	*;srp-GAL4,UAS-GFP/UAS-draper RNAi*^{HM501623}*;crq-GAL4,UAS-EGFP/+*
**Supplementary Fig. 2B', B''**	con/control	*y*^{1}*sc*v*^{1}*;srp-GAL4,UAS-EGFP/+;crq-GAL4,UAS-EGFP/+*
	RNAi/Draper RNAi	*y*^{1}*sc*v*^{1}*;srp-GAL4,UAS-EGFP/UAS-draper RNAi*^{HM501623}*;crq-GAL4,UAS-EGFP/+*
**Supplementary Fig. 2C**	control	*w;;crq-GAL4,UAS-nuclear red stinger*
	shark^{1}*/Df*	*w;shark*^{1}*/Df(2L)BSC434;crq-GAL4,UAS-nuclear red stinger*
**Supplementary Fig. 3A, B**	control	*w;srp-GAL4,UAS-EGFP/srp-GAL4,UAS-nuclear red stinger*
	draper$^{\Delta5}$	*w;srp-GAL4,UAS-EGFP/srp-GAL4,UAS-nuclear red stinger;draper*$^{\Delta5}$
	control	*w;;crq-GAL4,UAS-nuclear red stinger*
	src42^{E1}	*w;src42A*^{E1}*;crq-GAL4,UAS-nuclear red stinger*
	shark^{1}	*w;shark*^{1}*;crq-GAL4,UAS-nuclear red stinger*
**Supplementary Fig. 4A', A''**	*c1/c2/c3*	*w;;da-GAL4/+*
	wt1/wt2/wt3/*da>Draper-I*^{wt}	*w;+/UAS-Draper-I*^{wt}*;da-GAL4/+*
	m1/mt2/m3/*da>Draper-I*^{Y949F}	*w;+/UAS-Draper-I*^{Y949F}*;da-GAL4/+*
**Supplementary Fig. 4B**	control	*w*
	srp>Drpr-I^{wt}*;draper*$^{\Delta5}$	*w;srp-GAL4,UAS-draper-I*^{wt}*;draper*$^{\Delta5}$
	srp>Drpr-I^{Y949F}*;draper*$^{\Delta5}$	*w;srp-GAL4,UAS-draper-I*^{Y949F}*;draper*$^{\Delta5}$
**Supplementary Fig. 4C', C''**	control	*w;srp-GAL4,UAS-EGFP/+;crq-GAL4,UAS-EGFP/+*
	macrophages + *Drpr-I*^{wt}	*w;srp-GAL4,UAS-EGFP/UAS-Draper-I*^{wt}*;crq-GAL4,UAS-EGFP/+*
	macrophages + *Drpr-I*^{Y949F}	*w;srp-GAL4,UAS-EGFP/UAS-Draper-I*^{Y949F}*;crq-GAL4,UAS-EGFP/+*

**Supplementary Movie 1**	control	*w;;crq-GAL4,UAS-nuclear red stinger*
	src42^{E1}	*w;src42A*^{E1}*;crq-GAL4,UAS-nuclear red stinger*

Table S2, relates to experimental procedures. Sources of alleles and transgenes:

Allele:	Chromosome:	Obtained from:
srp-GAL4	2	Katja Bruckner, UCSF, USA
crq-GAL4	3	Brian Stramer, KCL, UK
pxn-GAL4	3	Brian Stramer, KCL, UK
da-Gal4	3	Bloomington Stock Centre, USA
UAS-nuclear red stinger	2 and 3	Brian Stramer, KCL, UK
w^{1118}	X	Bloomington Stock Centre, USA
$src42A^{E1}$	2	Bloomington Stock Centre, USA
$src42A^{K10108}$	2	Bloomington Stock Centre, USA
$src42A^{myri}$	2	Bloomington Stock Centre, USA
$shark^{1}$	2	Bloomington Stock Centre, USA
Df(2L)BSC434	2	Bloomington Stock Centre, USA
$draper^{\Delta 5}$	3	Paul Martin, University of Bristol, UK
UAS-draper RNAi TRiP line HM501623	2	Bloomington Stock Centre, USA
$UAS\text{-}src42A^{DN}$	3	David Strutt, University of Sheffield, UK
$UAS\text{-}draper\text{-}I^{wt}$	2	Eric Baehrecke, UMASS, USA
$UAS\text{-}draper\text{-}I^{Y949F}$ line 24127-5	2	Marc Freeman, UMASS, USA
UAS-draper-II line 03529-6	2	Marc Freeman, UMASS, USA
UAS-shark-2	X	Marc Freeman, UMASS, USA

Supplemental Experimental Procedures

Fly genetics

Precise genotypes of the embryos used in this study can be found in Table S1, with details on the sources of alleles/transgenes contained in Table S2.

Image processing and analysis

Z-stacks of fluorescently-labelled macrophages were despeckled and assembled into maximum projections using NIH ImageJ/Fiji [S1]. Macrophage movements in wound movies or movies of basal motility were tracked using the manual tracking plugin in ImageJ. To quantify overall behaviour of macrophages in movies following laser wounding, macrophage movements were measured with respect to the center of the wound over the course of the 20 min movie. The centre (centroid) of the wound was determined from brightfield/transmitted laser light images. Tracking enabled the net distance each macrophage moved towards or away from the center of the wound over the timecourse of the movie to be determined; macrophages were followed once they were within 60 µm of the wound's center. These measurements enabled the calculation of directionality towards the center of the wound (Cartesian distance from center of the wound at t = 0 min/net distance moved with respect to

the center of the wound) for each cell analysed. Directionality per macrophage was then averaged per embryo to give an overall measure of the wound response; only movies with wounds between 1000 and 2000 μm^2 at 10 min post-wounding were used in this dynamic analysis. For movies of basal motility speed was calculated by dividing the total distance moved over course of the movie by the time taken to move that distance.

To quantify macrophage wound responses from individual timepoints (20 or 60 min post-wounding), the number of macrophages in contact with/inside the wound edge was determined from z-stacks. Wound size was determined from brightfield images in ImageJ. The number of macrophages was then divided by the wound area and the value normalised according to the appropriate control average.

Z-stacks were also used to measure vacuolation (EGFP-negative inclusions within the macrophage cell body - only macrophages that were clearly distinct from their neighbours on the ventral midline were assessed to rule out the possibility of counting vacuoles from > 1 macrophage) or macrophage density (numbers on the ventral midline/cross-sectional area of the most dorsal confocal slice through the embryo). Maximum z-projections were used to measure macrophage spread area using the lasso tool in ImageJ. As per vacuolation analyses, only individual cells lying between the edges of the ventral nerve cord that could be distinguished from their neighbours were analysed, with > 30 cells quantified from at least 5 different embryos for each genotype.

To quantify Draper expression levels in vitro, the GFP channel of maximum projections was used to draw a mask around each GFP-positive macrophage. This masked selection was then used to measure the intensity of anti-Draper staining per cell and the spread area. The integrated density (intensity or mean gray value x area) was normalised to control levels to enable the comparison of Draper expression levels. All image analysis was carried out on blinded images and identical adjustments were made to contrast and brightness across experimental groups. All statistical analysis and production of graphs was carried out using Prism 6 (GraphPad).

Immunofluorescent staining of *Drosophila* embryos and macrophages in vitro

Embryos were fixed and immunostained as per Evans et al., 2010 [S2], using rabbit anti-LacZ (1:2000, Cappel), purified mouse anti-Fascin (1:100 clone sn7C, Developmental Studies Hybridoma Bank) or rabbit anti-pSrc (1:500, Invitrogen) as primary antibodies with goat anti-rabbit-AlexaFluor488 (Molecular probes), goat anti-mouse-FITC (Jackson Immunoresearch Laboratories) or goat anti-rabbit-AlexaFluor568 (Molecular Probes) used as secondary antibodies, respectively.

To probe Draper expression 14 stage 15 embryos were disaggregated in 70 μL 10 % FBS in Schneiders medium (Sigma) and allowed to adhere to Thermanox plastic coverslips (Nunc) for 2 h at room temperature in a humidified chamber. Macrophages were then fixed for 10 min in 4 % formaldehyde (Agar Scientific) in PBS. Macrophages were then permeabilised with 0.1 % Triton-X100 in PBS for 3 min and blocked with 2 % horse serum in PBS for 45 min. GFP-expressing macrophages were then stained using mouse anti-GFP (1:100, Abcam) and rabbit anti-Draper (1:500, kind gift from Marc Freeman [S3]) for 45 min in 2 % horse serum, with primary antibodies detected via goat anti-mouse-FITC and goat anti-rabbitAlexaFluor568 (both 1:200 in 2 % horse serum/PBS for 30 min), while Hoechst/NucBlue (Molecular Probes) was added as a nuclear dye. Cells were then mounted in Mowiol under a coverslip before confocal imaging on a Nikon A1 system using a 63X oil immersion lens.

Analysis of macrophage numbers per embryo

Embryos containing red stinger-expressing macrophages were fixed in 4 % formaldehyde according to standard fixation protocols [S2] and then cleared overnight in Dabco mountant at 4°C. Embryos were then transferred to slides in a minimal volume of Dabco and flattened under a coverslip in Mowiol mountant, such that macrophages were all in the same focal plane once the Mowiol had set. Embryos were then imaged on a Leica M205FA stereomicroscope fitted with a Leica DCF365 FX camera, with panoramic images of the whole embryo stitched together using the MosaicJ plugin [S4] in ImageJ. These images were

then blinded and macrophage numbers per embryo counted using red stinger expression and nuclear morphology to identify macrophages.

Western blotting of embryo lysates

30 stage 15/16 embryos were crushed and dissolved in 60 µl 2.5X sample buffer per sample, then boiled for 10 min at 95°C, before separation via SDS-PAGE on 7.5 % pre-cast gels (Bio-Rad). Standard wet transfer techniques were used to transfer separated proteins to nitrocellulose filters, which were subsequently blocked using 5 % BSA (Sigma) in TBS-Tween before incubation overnight at 4°C in rabbit anti-Draper [S3] or rabbit anti-αTubulin (Cell Signalling Technologies), with each diluted 1:1000 in blocking solution. After washing in 1 % BSA in TBS-Tween, goat anti-rabbit-HRP secondary antibodies (Dako) were used at a dilution of 1:6000 in 1 % BSA in TBS-Tween for 90 min at room temperature. After washing in TBS-T, secondary antibodies were detected using ECL select (Amersham). Blots were scanned and quantified by normalising the integrated density of Draper bands according to the corresponding αTubulin band after background subtraction. These values were then normalised according control (Figure S2) or Draper-I^{wt} levels (Figure S4) to reveal RNAi-mediated knockdown or compare expression with that of Draper-I^{Y949F}, respectively.

Macrophage sorting and qPCR

For sorting of macrophages, 50 embryos of each genotype were disaggregated, strained using a 40 µm mesh and resuspended in 500 µL Seecoff's buffer. Suspensions were then sorted using an Influx™ cell sorter with a 488nm Laser (BD Biosciences). Non-fluorescent embryos were used to set sort gates.

For quantification of RNAi-mediated knockdown of *draper-I* mRNA was extracted from sorted macrophages using RNAqueous-Micro Total RNA Isolation Kit (Ambion). For quantification of *drpr-I* expression in *drpr*$^{\Delta 5}$ mutant rescue experiments 50 embryos of each genotype were crushed in QIAzol Lysis Reagent (Qiagen) and RNA purified using an RNeasy Mini Kit (Qiagen). In both experiments RNA samples were then treated with DNase and RNA quantified using a NanoDrop 2000c (Thermo Scientific). Equal quantities of RNA were then

reverse transcribed using SuperScript III First-Strand Synthesis System (Invitrogen). Relative quantification of gene expression was carried out on a LightCycler 480 Real-Time PCR machine (Roche). The following Taqman assays (Applied Biosystems) were used: (i) Ribosomal protein L32 (ABI pre-made assay Dm02151827_g1) (ii) Draper-I custom assay, F-primer, TGTGATCATGGTTACGGAGGAC; R-primer, CAGCCGGGTGGGCAA; probe, CGCCTGCGATATAA as described in Logan et al., (2012) [S5]. Analysis was performed on $2^{-(\Delta\Delta Ct)}$ values.

Supplemental References

S1. Schindelin, J., Arganda-Carreras, I., Frise, E., Kaynig, V., Longair, M., Pietzsch, T., Preibisch, S., Rueden, C., Saalfeld, S., Schmid, B., et al. (2012). Fiji: an open-source platform for biological-image analysis. Nature methods 9, 676-682.

S2. Evans, I.R., Hu, N., Skaer, H., and Wood, W. (2010). Interdependence of macrophage migration and ventral nerve cord development in Drosophila embryos. Development 137, 1625-1633.

S3. Freeman, M.R., Delrow, J., Kim, J., Johnson, E., and Doe, C.Q. (2003). Unwrapping glial biology: Gcm target genes regulating glial development, diversification, and function. Neuron 38, 567-580.

S4. Thevenaz, P., and Unser, M. (2007). User-friendly semiautomated assembly of accurate image mosaics in microscopy. Microscopy research and technique 70, 135-146.

S5. Logan, M.A., Hackett, R., Doherty, J., Sheehan, A., Speese, S.D., and Freeman, M.R. (2012). Negative regulation of glial engulfment activity by Draper terminates glial responses to axon injury. Nature neuroscience 15, 722-730.
